# The E3 ubiquitin ligase IDOL suppresses PRRSV replication by targeting viral Nsp3 for SQSTM1-mediated selective autophagic degradation

**DOI:** 10.1128/mbio.01534-26

**Published:** 2026-09-15

**Authors:** Jiecong Yan, Yuhan Wu, Huirui Xu, Pengning Lai, Ling Huang, Zhan He, Yongsheng Xie, Chunhe Guo

**Affiliations:** 1 Guangdong Laboratory for Lingnan Modern Agriculture, State Key Laboratory for Animal Disease Control and Prevention, Key Laboratory of Zoonosis Prevention and Control of Guangdong Province, College of Veterinary Medicine, South China Agricultural University 12526 , Guangzhou, Guangdong, People's Republic of China; 2 School of Life Sciences and Environmental Resources, Yichun University 117771, Yichun, Jiangxi, People's Republic of China; Rutgers-Robert Wood Johnson Medical School, Piscataway, New Jersey, USA

**Keywords:** PRRSV, IDOL, Nsp3, ubiquitination, selective autophagy

## Abstract

Porcine reproductive and respiratory syndrome virus (PRRSV) is one of the most devastating pathogens affecting the global swine industry. Autophagy plays an important role in both host defense and PRRSV infection. However, the mechanisms by which the host exploits the autophagy pathway to antagonize PRRSV infection remain largely unknown. Here, we perform transcriptomic analysis of porcine alveolar macrophages isolated from PRRSV-infected piglets and reveal a significant upregulation of the mRNA levels for the E3 ubiquitin ligase IDOL. These findings are further validated by *in vitro* experiments. Functional studies reveal that IDOL acts as a host restriction factor for PRRSV: ectopic expression of IDOL markedly suppresses viral replication, while knockdown of endogenous IDOL enhances it. Mechanistically, IDOL catalyzes K63-linked polyubiquitination of the viral nonstructural protein 3 (Nsp3) at lysine 101 (K101) and recruits the selective autophagy receptor SQSTM1, thereby targeting Nsp3 for degradation via the autophagy-lysosomal pathway and effectively suppressing viral replication. Notably, the ubiquitin-associated and LC3-interacting region domains of SQSTM1 are essential for this autophagic clearance of Nsp3. Furthermore, using a reverse genetics approach, we generate an Nsp3 K101R mutant virus and confirm that this specific mutation enables the virus to evade IDOL-mediated Nsp3 degradation, thereby restoring its replication competence. In summary, we uncover an autophagy-dependent antiviral mechanism, establish IDOL as an innate immune regulator with a non-canonical function, and define a viral immune evasion strategy, positioning IDOL as a promising therapeutic target for PRRSV control.

**IMPORTANCE:**

Porcine reproductive and respiratory syndrome virus (PRRSV) represents a major threat to global swine production, inflicting substantial economic damage and endangering food security. Here, we identify the E3 ubiquitin ligase IDOL as a critical host restriction factor that combats PRRSV through a previously unrecognized autophagy-mediated mechanism. We demonstrate that PRRSV infection robustly upregulates the transcription factor ZNF460, which directly drives IDOL expression. During PRRSV infection, IDOL drives the autophagic degradation of Nsp3 by catalyzing its K63-linked ubiquitination at lysine 101 and engaging the selective autophagy receptor SQSTM1, triggering a potent antiviral response. Using reverse genetics, we generate an Nsp3 K101R mutant virus and demonstrate that this mutation enables the virus to escape IDOL-mediated degradation and restore replication competence, providing genetic validation of this antiviral axis. Collectively, our findings establish an autophagy-dependent antiviral mechanism, define IDOL as an intrinsic immune regulator with a previously unrecognized function, and elucidate a precise viral immune evasion strategy, thereby positioning IDOL as a promising therapeutic target for PRRSV intervention.

## INTRODUCTION

Porcine reproductive and respiratory syndrome virus (PRRSV) induces reproductive failure in sows and respiratory distress in piglets, establishing it as a critically damaging pathogen to the global swine industry (1, 2). The virus, an enveloped, single-stranded positive-sense RNA virus with a genome of approximately 15 kb encoding at least 10 open reading frames (ORFs), is a member of the *Arteriviridae* family within the order *Nidovirales* (3–7). Its replicase gene consists of two ORFs (ORF1a and ORF1b) that encode two distinct polyproteins (pp), pp1a and pp1ab (8, 9). These polyproteins are proteolytically cleaved by viral proteases into at least 16 mature, functional non-structural proteins (Nsps) (10–12). The majority of Nsps are implicated in viral replication, pathogenicity, and host immune suppression. These proteins assemble into a membrane-associated replication and transcription complex (RTC), which facilitates viral RNA synthesis and coordinates the transcription and replication processes (13–15). Specifically, Nsp2, Nsp3, and Nsp5 function as structural scaffolds within the RTC (15). ORFs 2–7 encode eight structural proteins: glycoprotein 2a (GP2a), envelope (E) protein, GP3, GP4, GP5, GP5a, matrix (M) protein, and nucleocapsid (N) protein (16, 17). Despite global efforts in vaccine development, inactivated vaccines fail to induce sufficiently effective neutralizing antibodies, while attenuated vaccines raise safety concerns. The persistent recombination and mutation of PRRSV further complicate disease control, underscoring the urgent need to identify effective antiviral protein targets for PRRSV prevention and management.

Autophagy is an essential physiological process in cells, involving the degradation and recycling of cellular components (18, 19). During autophagy, the cell forms a specialized double-membrane structure called an autophagosome (20), which engulfs damaged proteins and organelles and transports them to the lysosome for degradation (21–23). Autophagy serves as an efficient antiviral defense response by which host cells recognize and degrade viral components. A growing body of evidence demonstrates the interplay between viruses and autophagy in cellular antiviral defense (24). Autophagy exerts a dual role in PRRSV infection, either promoting or suppressing viral replication depending on the specific virus and the stage of the infection cycle. Research has demonstrated that PRRSV can induce autophagy to facilitate its own proliferation (25, 26). For instance, PRRSV Nsp2 disrupts Ca²^+^ homeostasis by inducing endoplasmic reticulum stress, ultimately activating autophagy via the CaMKII-AMPK-mTOR pathway to promote viral replication (25). The PRRSV envelope (E) protein induces SQSTM1-dependent autophagic degradation of DDX10, thereby antagonizing the antiviral activity of DDX10 (26). Conversely, host cells utilize autophagy to inhibit PRRSV infection (27–29). For instance, proteasome subunit beta type-1 (PSMB1) suppresses PRRSV replication by recruiting the neighbor of BRCA1 gene 1 protein (NBR1) to mediate the autophagic degradation of viral Nsp12 (27). Proteasome subunit beta type-4 (PSMB4) interacts with PRRSV Nsp1α, specifically targets residue K169 of Nsp1α for K63-linked ubiquitination, and induces the degradation of Nsp1α via the autophagy-lysosome pathway, consequently inhibiting PRRSV replication (28). In summary, autophagy plays a dual role in PRRSV infection, both promoting and suppressing viral replication; yet its mechanistic basis remains incompletely understood, with significant scientific questions still unresolved.

The E3 ubiquitin ligase inducible degrader of low-density lipoprotein receptor (IDOL), also known as MYLIP, was initially characterized as a protein containing an N-terminal four-point-one, ezrin, radixin, moesin (FERM) domain and a C-terminal RING-type zinc finger domain responsible for its ubiquitin ligase activity (30, 31). It is transcriptionally regulated by liver X receptors (LXRs) and plays a central role in cholesterol homeostasis by promoting the ubiquitination and subsequent degradation of the low-density lipoprotein receptor (LDLR) via the ubiquitin-proteasome pathway (30, 32). This activity downregulates cellular cholesterol uptake and helps maintain intracellular cholesterol balance (33, 34). Notably, beyond its well-established function in lipid metabolism, IDOL has also been implicated in neural development and function, as well as in the pathogenesis of neurodegenerative diseases, including Alzheimer’s disease (35).

Furthermore, emerging evidence indicates that IDOL-mediated lipid regulation contributes to host anti-infection immunity (36, 37). By triggering the degradation of LDLR family receptors, IDOL restricts their use as entry receptors by various pathogens, such as hepatitis C virus and intracellular bacteria (36). In addition, IDOL-driven cholesterol redistribution modulates inflammatory signaling in immune cells and alters the intracellular niche for pathogen survival, thereby enhancing innate immune defenses. To our knowledge, the role of IDOL in PRRSV infection has not yet been elucidated.

In this study, transcriptomic sequencing of alveolar macrophages isolated from PRRSV-infected piglets revealed that IDOL expression was significantly upregulated following viral infection. Mechanistically, IDOL inhibits viral replication by enhancing the interaction between the selective autophagy receptor SQSTM1 and the viral Nsp3 protein, thereby promoting Nsp3 degradation via the autophagy-lysosomal pathway. These findings provide important insights for the development of IDOL-targeted therapeutics against PRRSV and hold strategic significance for the control of PRRS.

## RESULTS

### PRRSV infection induces upregulation of IDOL expression

**Fig 1:**
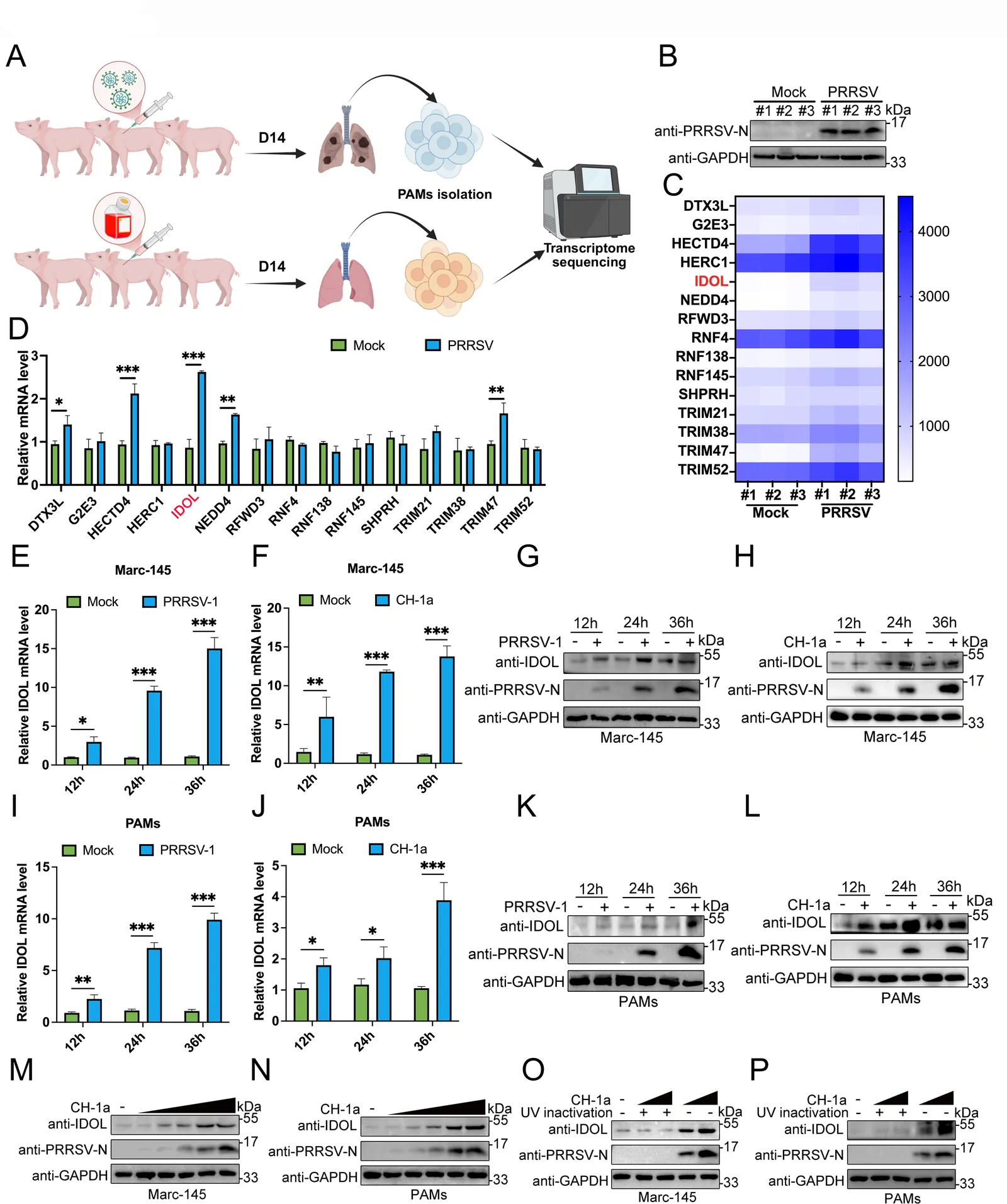
PRRSV infection induces upregulation of IDOL expression. (**A**) Schematic diagram of the piglet challenge experiment. (**B**) Analysis of N protein levels in cells from the infected and control groups by western blotting. (**C and D**) Heatmap presentation (**C**) and RT-qPCR validation (**D**) of 15 E3 ubiquitin ligase genes identified by transcriptomics in virus-infected piglets. (**E–L**) Time-dependent upregulation of IDOL. Marc-145 cells (**E–H**) or PAMs (**I–L**) were infected with the PRRSV-1 strain GD2025 or the PRRSV-2 strain CH-1a at an MOI of 0.5. Cells were harvested at 12, 24, and 36 hpi. IDOL mRNA levels were analyzed by RT-qPCR (**E, F, I, J**), and endogenous IDOL protein levels were analyzed by western blotting (**G, H, K, L**). (**M and N**) Dose-dependent upregulation of IDOL. Marc-145 cells (**M**) or PAMs (**N**) were infected with increasing doses of the CH-1a strain, and endogenous IDOL protein levels were assessed by western blotting. (**O and P**) Requirement for active viral replication. Marc-145 cells (**O**) or PAMs (**P**) were treated with either live or UV-inactivated CH-1a virus at the indicated doses. Endogenous IDOL protein levels were analyzed by western blotting. Note: Statistical comparisons (indicated by asterisks) are made between the PRRSV-infected group and the mock control at each corresponding time point. Results are representative of at least three independent biological replicates and are presented as mean ± SD (*n* = 3). *P < 0.05, **P < 0.01, and ***P < 0.001 denote statistical significance, ensuring the reproducibility of the findings.

To identify host antiviral genes capable of degrading PRRSV proteins, we performed an infection experiment in which piglets were challenged with PRRSV (2 × 10⁵ TCID₅₀/mL) alongside mock-infected controls. At 14 days post-infection, lung tissues were collected from both groups for the isolation of alveolar macrophages, which were subsequently subjected to transcriptome sequencing (Fig. 1A). Viral infection was successfully confirmed in cells derived from the challenged group, consistent with expected infection outcomes (Fig. 1B). Based on transcriptomic profiling, we selected 15 E3 ubiquitin ligase genes that exhibited the most pronounced upregulation at the transcriptional level following viral infection for further validation (Fig. 1C). RT-qPCR analysis confirmed that, although some genes did not reach statistical significance, the mRNA levels of these host factors generally paralleled the transcriptomic trends. Among them, IDOL displayed the most significant increase in mRNA expression, prompting us to hypothesize that it plays a pivotal role during PRRSV infection (Fig. 1D). To validate whether PRRSV infection upregulates IDOL expression, we conducted infection experiments using the PRRSV-1 strain GD2025 and the PRRSV-2 strain CH-1a (which was used as the default strain in subsequent experiments unless otherwise specified). Porcine alveolar macrophages (PAMs) and Marc-145 cells were infected with these viruses, respectively. The results demonstrated a progressive, time-dependent increase in both IDOL mRNA and protein levels during infection (Fig. 1E through L). To further investigate the induction of IDOL during early PRRSV infection, we examined IDOL expression at 3, 6, and 9 hpi. The results revealed that IDOL was upregulated at the early stages of infection, indicating that IDOL is induced as an early host response to PRRSV infection (Fig. S1A through D). We further examined the relationship between viral dose and host response by infecting Marc-145 and PAMs with increasing doses of PRRSV. IDOL protein levels exhibited a dose-dependent increase (Fig. 1M and N). This dose-dependent upregulation prompted us to test its specificity. When cells were treated with inactivated virus, no increase in IDOL was observed (Fig. 1O and P), confirming that the induction of IDOL is a specific consequence of active viral replication, not merely exposure to viral particles.

To investigate the transcriptional regulation of IDOL, we cloned a 2000-bp promoter fragment of the IDOL gene along with a series of truncations (M1–M8) into the pGL6 luciferase reporter vector. Luciferase activity was abolished in constructs M5 through M8, narrowing the core promoter region to −125 to −1 bp (Fig. S1E). Using the JASPAR vertebrate database (38), we predicted potential binding sites within this core promoter for four transcription factors: KLF15, ZNF263, ZNF460, and SP4 (Fig. S1F). In order to identify transcription factors regulating the IDOL promoter, we measured the mRNA levels of candidate factors following viral infection. Quantitative analysis revealed that PRRSV infection significantly upregulated the expression of the transcription factors KLF15 and ZNF460 (Fig. S1G). Next, we overexpressed each factor in Marc-145 cells and measured IDOL expression via RT-qPCR. Overexpression of ZNF460 resulted in a significant increase in IDOL mRNA (Fig. S1H). Conversely, siRNA-mediated knockdown of ZNF460 led to a corresponding decrease in IDOL transcript levels (Fig. S1I), confirming that ZNF460 positively regulates IDOL expression. To determine the transcriptional regulation of IDOL by ZNF460, we co-transfected cells with the IDOL promoter dual-luciferase reporter vector along with either a ZNF460 expression plasmid or an empty vector control. The dual-luciferase assay demonstrated that ZNF460 expression significantly upregulated the activity of the IDOL promoter (Fig. S1J). In addition, to further investigate the interaction, we performed chromatin immunoprecipitation (ChIP) assays using ZNF460-Myc to immunoprecipitate the core promoter region of IDOL. The results demonstrated that ZNF460 binds to the IDOL promoter (Fig. S1K), demonstrating its direct involvement in regulating IDOL expression. Together, these findings demonstrate that PRRSV infection upregulates IDOL expression through a ZNF460-dependent transcriptional mechanism.

### IDOL exhibits antiviral activity against PRRSV

**Fig 2:**
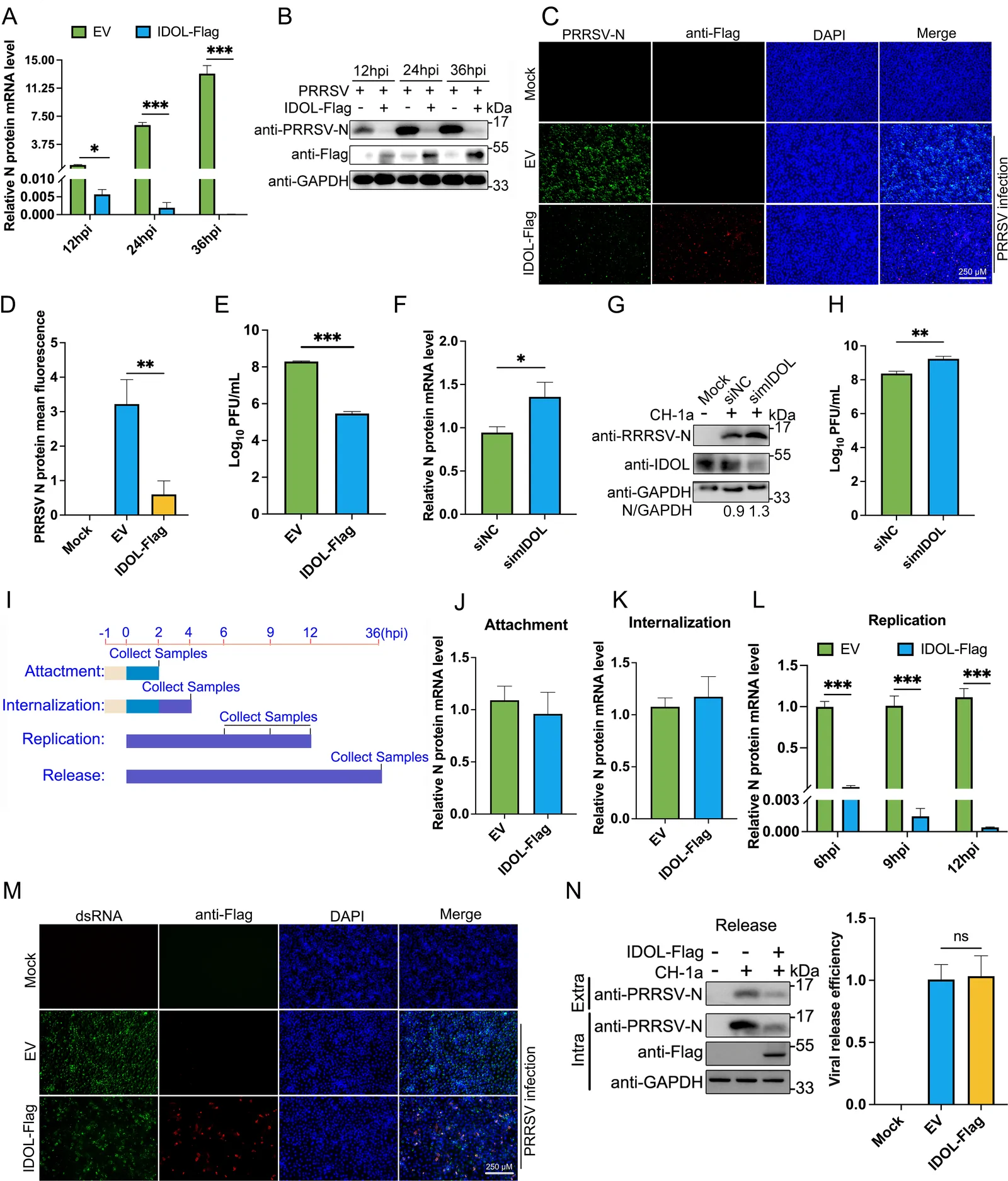
IDOL exhibits antiviral activity against PRRSV. (**A and B**) Marc-145 cells transfected with IDOL-Flag or vector were infected (MOI = 0.5) and harvested at the indicated times for viral RNA (RT-qPCR) (**A**) and protein (western blotting) (**B**) analysis. (**C and D**) Similarly, transfected cells were analyzed at 24 hpi by IFA (**C**), and the fluorescence intensity of the N protein quantified using ImageJ software (**D**). (**E**) Viral titers were determined by plaque assay. (**F–H**) After transfection with simIDOL-2 or NC siRNA for 24 h, Marc-145 cells were infected with the CH-1a strain (MOI  =  0.5) and analyzed at 24 hpi by RT-qPCR (**F**), Western blotting (**G**), and plaque assay (**H**). (**I**) Schematic diagram of the experimental strategy for assessing the role of IDOL in the PRRSV life cycle. (**J and K**) Viral attachment (**J**) and internalization (**K**) were assessed in transfected Marc-145 cells infected with PRRSV (MOI = 10). Cells were incubated at 4°C for 2 h to allow binding; for internalization, unbound virus was then removed prior to a 2-h incubation at 37°C. N gene mRNA levels were measured by RT-qPCR. (**L**) Marc-145 cells transfected as above were infected with PRRSV (MOI  =  0.5) and collected at 6, 9, and 12 hpi for RT-qPCR. Note: Statistical comparisons (indicated by asterisks) are between the indicated groups at each time point. (**M**) Similarly treated cells were fixed at 18 hpi for immunostaining of dsRNA. (**N**) Cells and supernatants from identically transfected and infected cultures were harvested at 36 hpi, and N protein levels in lysates and supernatants were assessed by western blotting and quantified by ImageJ software. Viral release efficiency was calculated as the ratio of extracellular to intracellular N protein levels (EC/IC). Results are representative of at least three independent biological replicates and are presented as mean ± SD (*n* = 3). *P < 0.05, **P < 0.01, and ***P < 0.001 denote statistical significance, ensuring the reproducibility of the findings.

To examine the role of IDOL during PRRSV infection, Marc-145 cells were transfected with either an IDOL-expressing plasmid or an empty vector control. At 24 h post-transfection, cells were infected with PRRSV at an MOI of 0.5. Cellular samples and supernatants were harvested at designated time points to evaluate PRRSV N protein expression and viral load. Both mRNA and protein levels of PRRSV N were significantly decreased at 12, 24, and 36 h post-infection (hpi) in IDOL-transfected cells relative to the control group (Fig. 2A and B). Furthermore, at 24 h post-infection, we examined the impact of IDOL on PRRSV infection using immunofluorescence assays (IFA, Fig. 2C). Quantitative analysis of the viral N protein fluorescence intensity with ImageJ software revealed that IDOL significantly suppressed PRRSV infection (Fig. 2D). Consistently, plaque assays also showed a marked reduction in viral titer (Fig. 2E). These findings suggest that IDOL exerts an antiviral effect against PRRSV.

To validate this observation, we designed two African green monkey-specific siRNAs targeting IDOL for knockdown in Marc-145 cells. As simIDOL-2 exhibited the highest knockdown efficiency (Fig. S2A), it was selected for all subsequent experiments. Compared to the negative control, IDOL knockdown led to increased accumulation of viral RNA, elevated N protein synthesis, and higher viral titers (Fig. 2F through H). Given that PAMs are the primary target cells for PRRSV infection, we synthesized three porcine-specific siRNAs targeting IDOL to definitively assess its impact on viral replication. Two of these, sipIDOL-2 and sipIDOL-3, showed substantial knockdown efficacy (Fig. S2B). To achieve maximal knockdown, we used sipIDOL-2 and sipIDOL-3 in combination, which reduced IDOL expression to nearly undetectable levels. Under these efficient knockdown conditions, subsequent experiments in PAMs revealed that IDOL depletion significantly increased viral RNA accumulation, N protein synthesis, and viral titers compared to the negative control (Fig. S2C through E). Given the marked differences between primary macrophages and immortalized cell lines in PRRSV susceptibility and antiviral responses, we compared the endogenous IDOL expression in Marc-145 cells and PAMs under physiological conditions. The results showed that endogenous IDOL levels were largely comparable between the two cell types, with PAMs exhibiting slightly lower IDOL protein abundance (Fig. S2F). These findings indicate that IDOL functions as an antiviral protein capable of effectively restricting PRRSV infection. Our next objective is to determine which stage of the viral life cycle is targeted by IDOL to exert its antiviral activity.

To evaluate the role of IDOL in the PRRSV life cycle, we assessed the effect of IDOL overexpression on viral adsorption, entry, replication, and release (Fig. 2I). Marc-145 cells transfected with IDOL-Flag or empty vector were inoculated with PRRSV at an MOI of 10 and incubated at 4°C for 2 h to facilitate viral attachment. Unbound virus particles were then removed by washing, and cell samples were analyzed via RT-qPCR, measuring N protein mRNA to evaluate attachment. IDOL overexpression did not significantly affect viral adsorption compared to the control (Fig. 2J). To examine internalization, transfected cells were infected with PRRSV under the same conditions, followed by incubation at 4°C for 2 h and then at 37°C for an additional 2 h. RT-qPCR analysis revealed that IDOL also had no significant effect on viral internalization (Fig. 2K). For the replication stage, cells were harvested at 6, 9, and 12 hpi for RT-qPCR. PRRSV N mRNA levels were significantly reduced in IDOL-overexpressing cells at all time points, indicating strong inhibition of viral replication (Fig. 2L). To confirm this, we transfected Marc-145 cells with IDOL-Flag or empty vector, infected them with PRRSV, and performed an IFA at 18 hpi to detect dsRNA. IDOL overexpression markedly reduced dsRNA levels, further supporting its role in suppressing replication (Fig. 2M). We next investigated the effect of IDOL knockdown in PAMs on the PRRSV replication stage. Quantitative analysis revealed that IDOL depletion facilitated viral replication (Fig. S2G). To assess viral release, cells were infected at an MOI of 0.5 for 36 h, and viral N protein levels in cell lysates and supernatants were analyzed by western blotting. IDOL did not influence viral release (Fig. 2N). In conclusion, IDOL functions as an antiviral protein, exerting its antiviral effect through the specific suppression of the PRRSV replication phase.

### IDOL can target and degrade viral Nsp3 through autophagy

**Fig 3:**
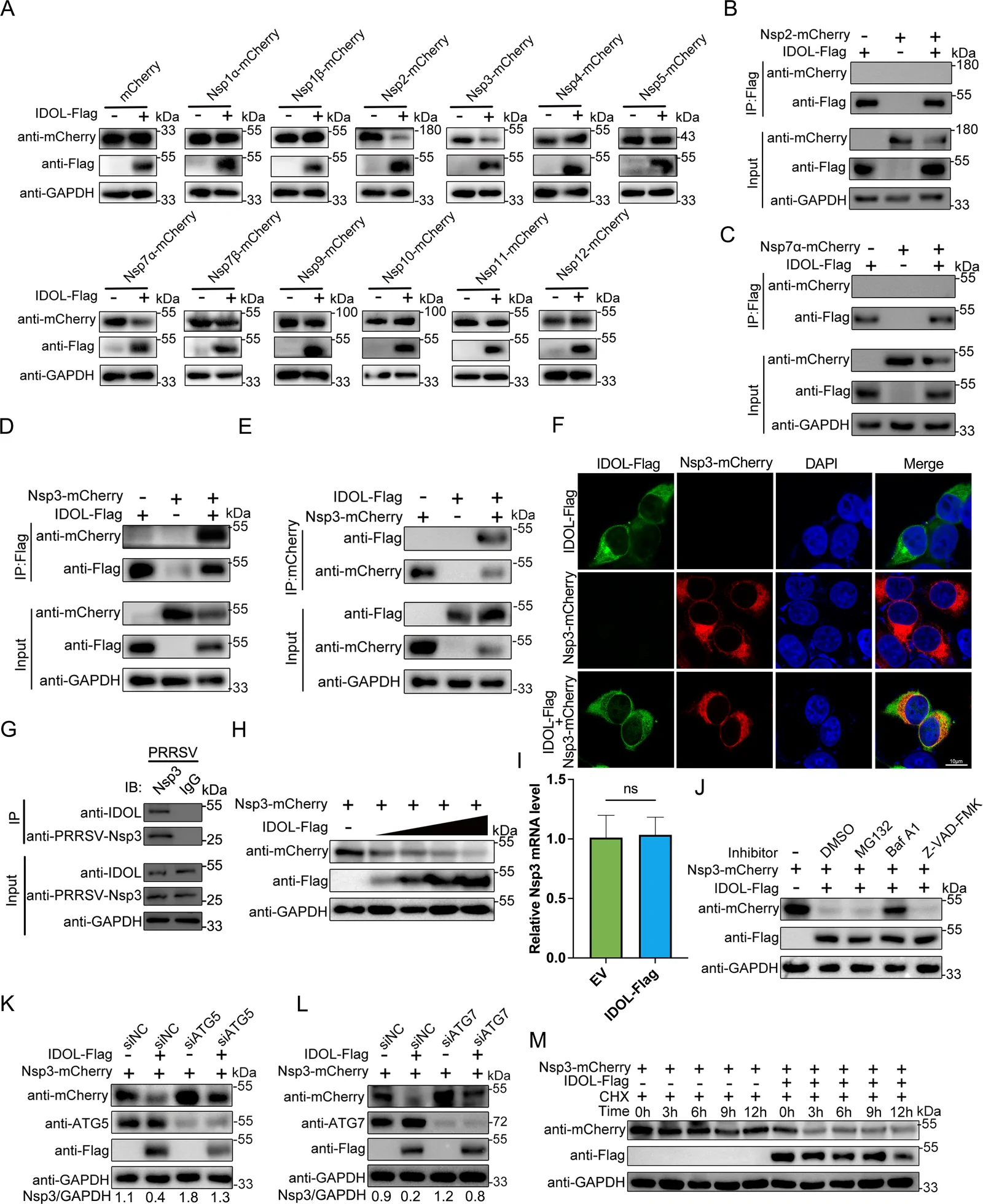
IDOL can target and degrade viral Nsp3 through autophagy. (**A**) HEK 293T cells were cotransfected with IDOL-Flag or empty vector and various mCherry-tagged PRRSV Nsp plasmids. Nsp levels were assessed by western blotting 24 h later. (**B and C**) HEK 293T cells were cotransfected with IDOL-Flag or empty vector together with Nsp2-mCherry (**B**) or Nsp7α-mCherry (**C**). Co-IP was performed using an anti-Flag antibody 24 h post-transfection, followed by western blotting analysis. (**D and E**) HEK 293T cells were co-transfected with IDOL-Flag or empty vector and Nsp3-mCherry. Co-IP was performed using anti-Flag (**D**) or anti-mCherry (**E**) antibodies, followed by western blotting analysis. (**F**) HEK 293T cells were cotransfected with IDOL-Flag and Nsp3-mCherry. Cells were fixed 24 h post-transfection and analyzed by confocal microscopy for co-localization. (**G**) To verify the interaction between IDOL and Nsp3, PAMs were infected with PRRSV at an MOI of 0.5, and cell samples were collected for Co-IP assays. (**H**) HEK 293T cells were cotransfected with increasing amounts of IDOL-Flag (0.5, 1, 1.5, and 2 μg) and Nsp3-mCherry. Cell lysates were analyzed by western blotting 24 h post-transfection to assess Nsp3 protein levels. (**I**) HEK 293T cells were cotransfected with IDOL-Flag or empty vector and Nsp3-mCherry. Total RNA was extracted 24 h post-transfection and subjected to RT-qPCR to measure Nsp3 mRNA levels. (**J**) HEK 293T cells were cotransfected with IDOL-Flag or empty vector and Nsp3-mCherry. At 12 h post-transfection, cells were treated with DMSO, MG132 (10 μM), Baf A1 (1 μM), or Z-VAD-FMK (10 μM) for 12 h, followed by western blotting analysis of Nsp3 protein levels. (**K and L**) Western blotting analysis of Nsp3 in cells transfected with siATG5 (**K**) or siATG7 (**L**) followed by cotransfection with IDOL-Flag and Nsp3-mCherry. (**M**) CHX chase assay: cells coexpressing IDOL-Flag and Nsp3-mCherry were treated with CHX (50 μg/mL) and harvested at the indicated times for western blotting. Note: To minimize antibody heavy/light-chain interference in Co-IP assays, measures including cross-species antibody usage were employed, although some background interference remained. Results are representative of at least three independent biological replicates and are presented as mean ± SD (*n* = 3). *P < 0.05, **P < 0.01, and ***P < 0.001 denote statistical significance, ensuring the reproducibility of the findings.

It is well established that most Nsps of PRRSV participate in viral transcription and replication (39). E3 ubiquitin ligases recognize specific substrates and catalyze the attachment of ubiquitin molecules, thereby targeting them for degradation through the ubiquitin-proteasome system or selective autophagy (40–42). Given that the E3 ubiquitin ligase IDOL can inhibit viral replication, we hypothesized that IDOL may facilitate the degradation of specific PRRSV Nsps. To test this hypothesis, we co-transfected HEK 293T cells with IDOL and plasmids encoding various viral Nsps and assessed the effect of IDOL on their expression. IDOL downregulated the expression of Nsp2, Nsp3, and Nsp7α (Fig. 3A). We next investigated whether IDOL interacts with Nsp2, Nsp3, and Nsp7α. Co-immunoprecipitation assays were performed using an IDOL-Flag plasmid together with Nsp2, Nsp3, or Nsp7α. The results showed that IDOL did not interact with Nsp2 or Nsp7α (Fig. 3B and C), but exhibited a strong interaction with Nsp3 (Fig. 3D and E). Next, confocal microscopy was employed to assess the co-localization of IDOL and Nsp3. The results demonstrated clear co-localization of Nsp3 with IDOL in the cytoplasm (Fig. 3F). Meanwhile, we infected PAMs with PRRSV and performed Co-IP using an anti-PRRSV Nsp3 antibody to validate the interaction between IDOL and Nsp3. The results were consistent with the expected interaction (Fig. 3G). To further validate the interaction between Nsp3 and IDOL under physiological conditions, Co-IP was performed on lung tissue samples from PRRSV-infected and mock-infected piglets. In the input samples, PRRSV infection was confirmed in the infected group, and IDOL expression was significantly upregulated in PRRSV-infected piglets (Fig. S3A). In the IP samples, an interaction between Nsp3 and IDOL was detected (Fig. S3A).

Given that IDOL specifically interacted with Nsp3 and exerted the most pronounced downregulatory effect on this protein, we focused on elucidating the mechanism by which IDOL regulates Nsp3 expression. To this end, we co-transfected increasing amounts of IDOL-Flag with Nsp3 into HEK 293T cells and assessed Nsp3 degradation efficiency. The results showed that IDOL reduced Nsp3 protein levels in a dose-dependent manner (Fig. 3H). To determine whether this downregulation occurred at the transcriptional level, we performed RT-qPCR 24 h after co-transfection of IDOL-Flag and Nsp3. The results indicated that IDOL did not affect Nsp3 mRNA levels (Fig. 3I). To further elucidate the underlying mechanism, cells co-expressing IDOL-Flag and Nsp3 were treated with the proteasome inhibitor MG132, the autophagy inhibitor Bafilomycin A1 (Baf A1), or the apoptosis inhibitor Z-VAD-FMK. Notably, Nsp3 protein levels were restored to control levels upon Baf A1 treatment, indicating that IDOL promotes Nsp3 degradation via the autophagy-lysosomal pathway (Fig. 3J). To confirm that Nsp3 is degraded via the autophagolysosomal pathway, we examined the effect of IDOL on Nsp3 degradation after knocking down the essential autophagy genes ATG5 and ATG7. The results showed that knockdown of either ATG5 or ATG7 markedly blocked IDOL-driven degradation of Nsp3 (Fig. 3K and L). These data collectively indicate that IDOL-mediated Nsp3 degradation is autophagy-dependent. Furthermore, in cells treated with cycloheximide (CHX) to inhibit *de novo* protein synthesis, IDOL overexpression significantly shortened the half-life of Nsp3 (Fig. 3M), confirming that IDOL accelerates Nsp3 degradation. To further characterize the temporal dynamics of IDOL-mediated Nsp3 degradation, we overexpressed IDOL in Marc-145 cells, infected them with PRRSV, and collected samples at 24, 36, 48, and 60 hpi to assess Nsp3 protein abundance. Compared with the control group, IDOL-mediated Nsp3 degradation was clearly detectable at 24 hpi and persisted throughout the late stages of infection (Fig. S3B). Interestingly, Nsp3 levels also gradually declined in the control group during late infection, which may be attributable to reduced expression of replication-associated non-structural proteins once viral replication had peaked. In addition, we found that the antiviral effect of IDOL remained significant at 60 hpi, indicating sustained antiviral activity (Fig. S3B).

### IDOL suppresses viral replication through its essential enzymatic activity

**Fig 4:**
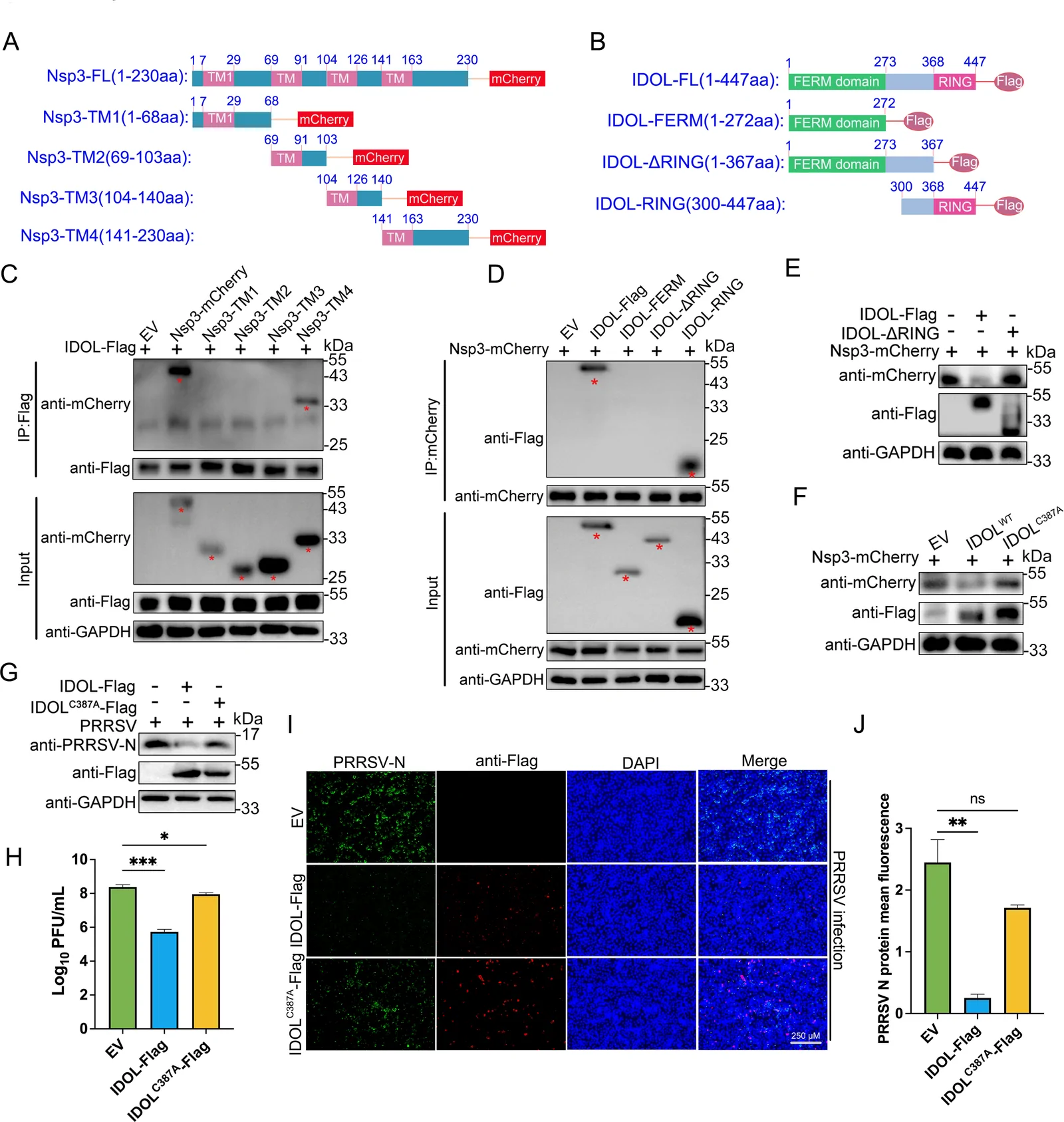
IDOL suppresses viral replication through its essential enzymatic activity. (**A and B**) Schematic representation of truncated mutants of Nsp3 (**A**) and IDOL (**B**) used in this study. (**C and D**) HEK 293T cells were co-transfected with full-length IDOL and truncated Nsp3 mutants (**C**), or with full-length Nsp3 and truncated IDOL mutants (**D**); cells were harvested 24 h later for Co-IP and western blotting. (**E and F**) HEK 293T cells were co-transfected with Nsp3-mCherry together with IDOL-Flag, IDOL-ΔRING (**E**), IDOL^C387A^-Flag (**F**), or corresponding empty vector controls. Cell lysates were subjected to western blotting analysis 24 h after transfection. (**G–J**) Marc-145 cells were transfected with IDOL-Flag, IDOL^C387A^-Flag, or empty vector for 24 h, followed by infection with the CH-1a strain (MOI = 0.5). At 24 hpi, cells and culture supernatants were collected for analysis by western blotting (**G**), plaque assay (**H**), IFA (**I**), and fluorescence intensity analysis (**J**). The asterisk indicates the position of the target protein band. Results are representative of at least three independent biological replicates and are presented as mean ± SD (*n* = 3). *P < 0.05, **P < 0.01, and ***P < 0.001 denote statistical significance, ensuring the reproducibility of the findings.

To identify the key domain of Nsp3 responsible for its interaction with IDOL, we performed domain prediction using DeepTMHMM (43), which reveals that Nsp3 contains four transmembrane domains. Accordingly, we constructed four truncated versions of Nsp3 and found that the TM4 domain is essential for the interaction with IDOL (Fig. 4A and C). IDOL contains an N-terminal FERM domain and a C-terminal RING domain, separated by a short linker region (44). Co-IP assays using various IDOL truncations together with Nsp3-mCherry showed that Nsp3 specifically interacted with the RING domain of IDOL, which is responsible for its ubiquitin ligase activity (Fig. 4B and D). These results led us to hypothesize that IDOL-mediated degradation of Nsp3 depends on its E3 ubiquitin ligase activity. The IDOL mutant lacking the RING domain (IDOL-∆RING) failed to degrade Nsp3 (Fig. 4E). Similarly, the catalytically inactive mutant IDOL^C387A^ also lost the ability to promote Nsp3 degradation (Fig. 4F). Given the critical role of IDOL’s ubiquitin ligase activity in mediating Nsp3 degradation, we investigated whether its antiviral function depends on this enzymatic activity. To test this hypothesis, Marc-145 cells were transfected with pCAGGS-Flag (empty vector), IDOL-Flag, or the catalytically inactive mutant IDOL^C387A^-Flag, followed by PRRSV infection. At 24 hpi, samples were collected and analyzed by western blotting, IFA, and plaque assays. Notably, cells expressing IDOL^C387A^ exhibited higher levels of N protein and increased viral titers compared to those expressing wild-type IDOL. However, the catalytic mutant still retained partial antiviral activity relative to the empty vector control (Fig. 4G through J).

### IDOL facilitates the polyubiquitination of Nsp3 at lysine residue 101

**Fig 5:**
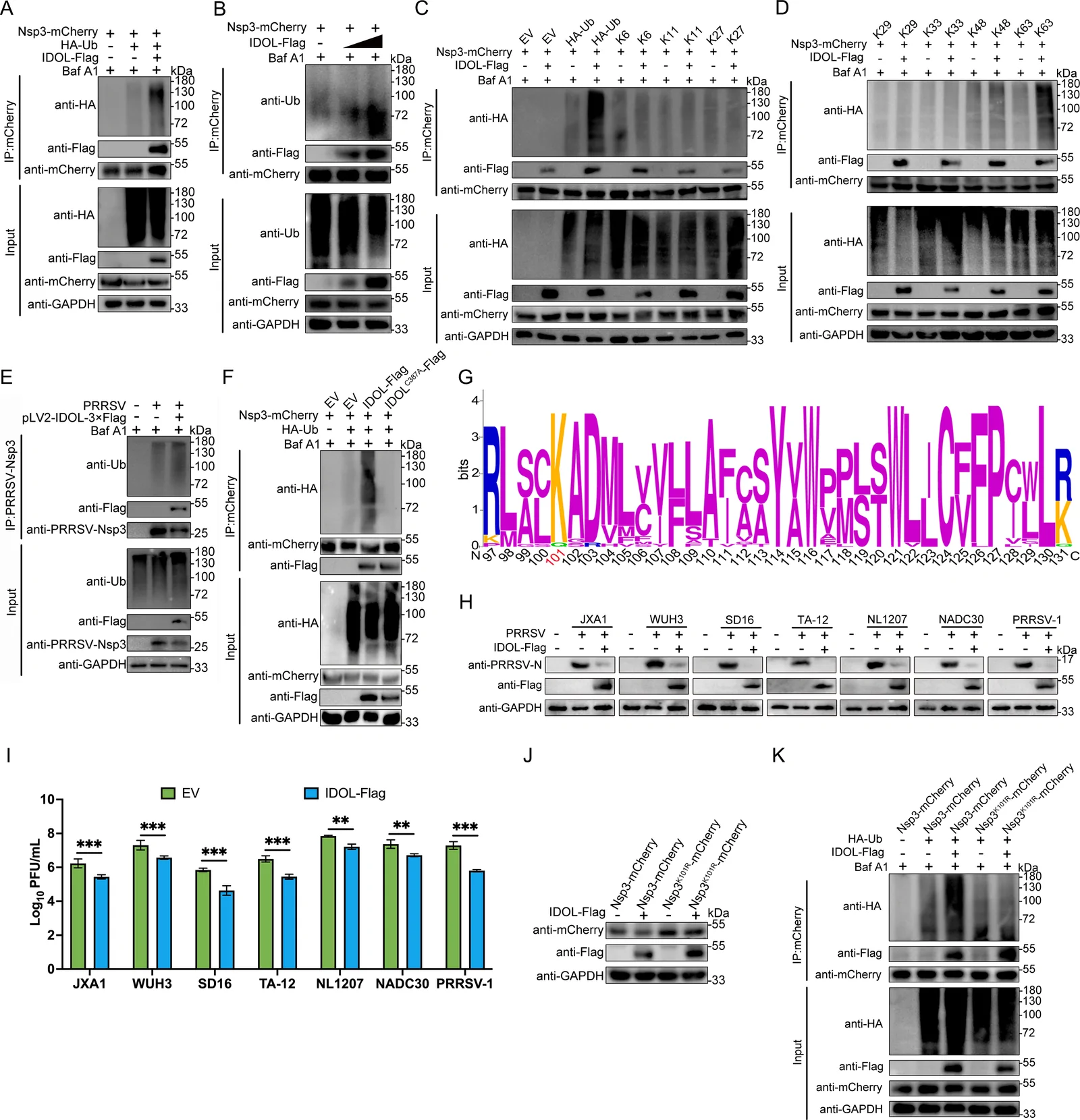
IDOL facilitates the polyubiquitination of Nsp3 at lysine residue 101. (**A**) Ubiquitination of Nsp3-mCherry by IDOL-Flag or empty vector co-expressed with HA-Ub, analyzed by anti-mCherry Co-IP and western blotting. (**B**) Endogenous ubiquitination of Nsp3-mCherry in cells expressing IDOL-Flag or empty vector, assessed by Co-IP. (**C and D**) Ubiquitin linkage analysis: Nsp3-mCherry and IDOL-Flag were co-expressed with HA-tagged wild-type or mutant ubiquitin (K6, K11, K27, K29, K33, K48, K63), followed by Co-IP. (**E**) PAMs were infected with an IDOL lentiviral vector and subsequently inoculated with PRRSV, and Co-IP assays were performed to examine the ubiquitination level of Nsp3. (**F**) Nsp3 ubiquitination in cells expressing IDOL-Flag, catalytically inactive IDOL^C387A^-Flag, or empty vector. (**G**) Sequence alignment of Nsp3 from PRRSV-1 and PRRSV-2 strains via Weblogo3, highlighting K101 conservation. (**H and I**) Antiviral activity across strains: Marc-145 cells transfected with IDOL-Flag or empty vector were infected with different PRRSV strains and analyzed by western blotting (**H**) and plaque assay (**I**). (**J**) HEK 293T cells were co-transfected with IDOL-Flag and Nsp3-mCherry or Nsp3^K101R^-mCherry. Protein levels were analyzed by western blotting at 24 h post-transfection. (**K**) Ubiquitination of Nsp3 or Nsp3^K101R^ in cells co-expressing HA-Ub and IDOL-Flag, assessed by Co-IP. Note: To minimize antibody heavy/light-chain interference in Co-IP assays, measures including cross-species antibody usage were employed, although some background interference remained. Results are representative of at least three independent biological replicates and are presented as mean ± SD (*n* = 3). *P < 0.05, **P < 0.01, and ***P < 0.001 denote statistical significance, ensuring the reproducibility of the findings.

Ubiquitination serves as a critical regulator of protein stability by targeting substrates for proteasomal degradation or autophagic clearance (41, 42). E3 ubiquitin ligases determine substrate specificity by catalyzing the transfer of ubiquitin to target proteins, leading to their degradation through the ubiquitin-proteasome system (UPS) or selective autophagy pathways (45, 46). To investigate whether IDOL mediates the ubiquitination of Nsp3, we examined the extent of polyubiquitin conjugation on Nsp3 in the presence or absence of IDOL. Our results showed that IDOL overexpression enhanced the polyubiquitination of Nsp3 (Fig. 5A and B). Given that different types of polyubiquitin linkages on lysine residues of substrate proteins can elicit distinct biological outcomes, we further characterized the topology of ubiquitin chains conjugated to Nsp3 in the presence of IDOL. IDOL was found to primarily catalyze K63-linked polyubiquitination of Nsp3 (Fig. 5C and D). To further verify whether IDOL promotes Nsp3 ubiquitination under physiological conditions, we constructed a lentiviral vector for IDOL overexpression, packaged the lentivirus, and infected PAMs to overexpress IDOL. The cells were then inoculated with PRRSV, and the ubiquitination of endogenous Nsp3 was subsequently examined. The results showed that IDOL increased the ubiquitination levels of endogenous Nsp3 (Fig. 5E). These findings suggest that IDOL may facilitate the reduction of Nsp3 levels by promoting its ubiquitination. In line with these results, we previously demonstrated that the RING-deletion mutant IDOL-∆RING, as well as the catalytically inactive mutant IDOL^C387A^, both lost the ability to degrade Nsp3 (Fig. 4E and F). Together, these data support the conclusion that IDOL induces ubiquitin-dependent degradation of Nsp3, largely through K63-linked polyubiquitin chains. We therefore further investigated whether the enzymatic activity of IDOL also plays an essential role in catalyzing the ubiquitination of Nsp3. As expected, the IDOL^C387A^ mutant failed to promote the ubiquitination of Nsp3 (Fig. 5F).

To identify the specific lysine residue(s) targeted by IDOL for polyubiquitination, we analyzed and compared the amino acid sequences of Nsp3 from 15 representative PRRSV-1 strains and 15 representative PRRSV-2 strains (the genetic accession numbers of these strains are summarized in Table S1). Sequence analysis revealed that lysine is conserved at position 101 of Nsp3 in the vast majority of strains, with mutations observed in only a small subset. We speculated that these rare mutations may represent adaptive changes acquired during viral evolution (Fig. 5G). We then evaluated the antiviral effect of IDOL across different PRRSV strains and found that IDOL significantly reduced both N protein expression and viral titers in all tested strains (Fig. 5H and I). These observations led us to hypothesize that Lys101 of Nsp3 is the primary site for IDOL-mediated ubiquitination. To test this hypothesis, we generated a mutant plasmid, Nsp3^K101R^-mCherry, by substituting lysine 101 with arginine using site-directed mutagenesis. We first assessed whether IDOL could still degrade the Nsp3^K101R^ mutant. While overexpression of IDOL markedly reduced the protein level of wild-type Nsp3 (Nsp3^WT^), it had no significant effect on the level of Nsp3^K101R^ (Fig. 5J). We next evaluated the ubiquitination of Nsp3^K101R^ by IDOL. Results showed that, compared to Nsp3^WT^, the conjugation of ubiquitin chains to Nsp3^K101R^ was substantially reduced in IDOL-expressing cells (Fig. 5K). Taken together, these data demonstrate that IDOL mediates Nsp3 degradation through K63-linked ubiquitination at Lys101 in a manner dependent on its E3 ubiquitin ligase activity.

### IDOL recruits SQSTM1 to mediate the selective autophagic degradation of Nsp3

**Fig 6:**
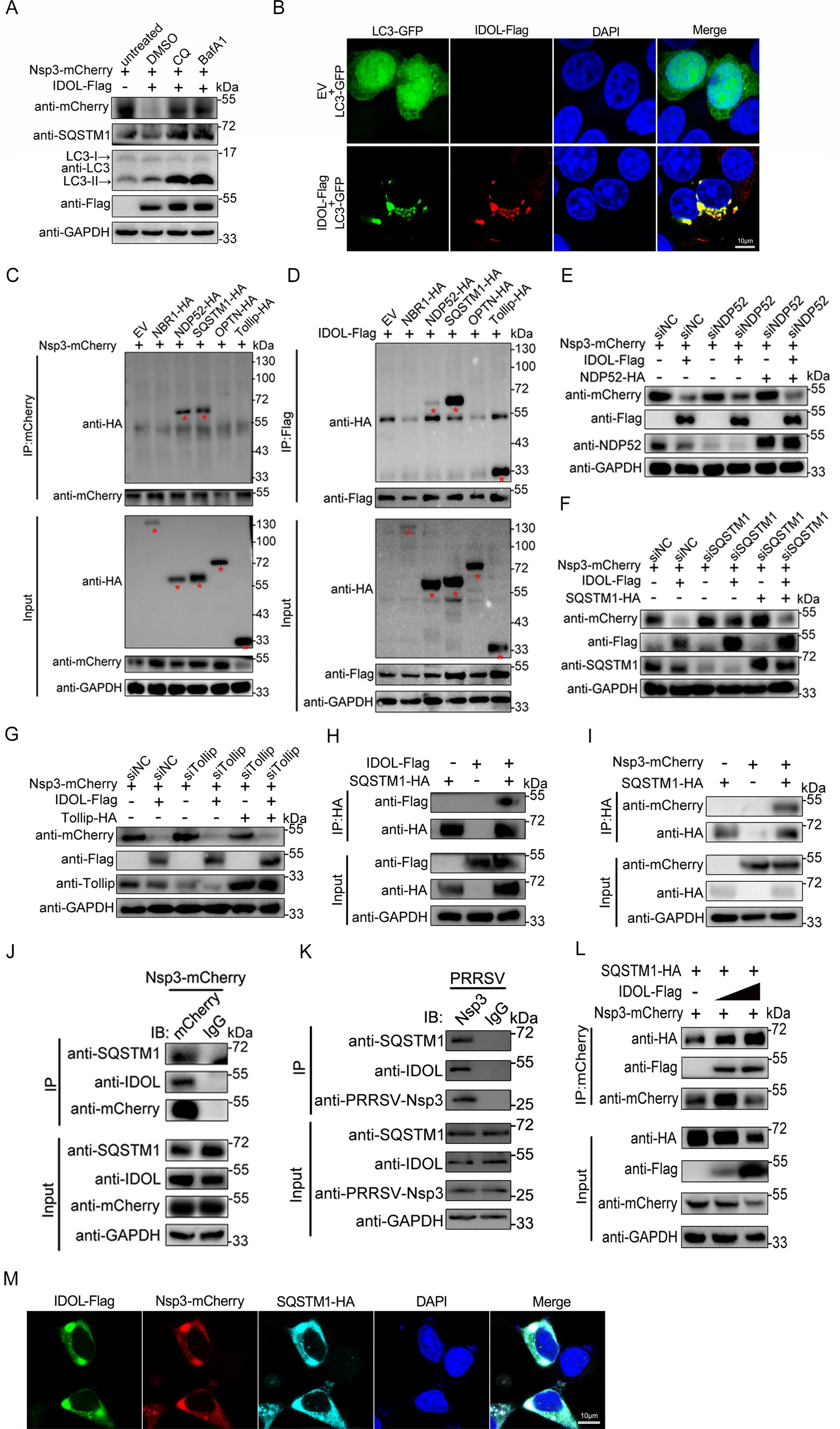
IDOL recruits SQSTM1 to mediate the selective autophagic degradation of Nsp3. (**A**) HEK 293T cells were co-transfected with Nsp3-mCherry and IDOL-Flag or empty vector. At 12 h post-transfection, cells were treated with DMSO, CQ (10 μM), or Baf A1 (1 μM) for 12 h, followed by western blotting. (**B**) Induction of autophagic flux assessed by LC3-GFP puncta formation upon IDOL expression. (**C and D**) Screening of Nsp3 (**C**) or IDOL (**D**) interactions with HA-tagged autophagy receptors by Co-IP. (**E–G**) HEK 293T cells were transfected with siNDP52 (**E**), siSQSTM1 (**F**), siTOLLIP (**G**), or NC siRNA for 24 h, followed by co-transfection with Nsp3-mCherry, IDOL-Flag, and NDP52-HA (**E**), SQSTM1-HA (**F**), or TOLLIP-HA (**G**). Cell lysates were analyzed by western blotting. (**H and I**) Confirmation of direct interaction between SQSTM1-HA and IDOL-Flag (**H**) or Nsp3-mCherry (**I**) by Co-IP. (**J**) HEK 293T cells expressing Nsp3-mCherry were subjected to Co-IP using an anti-mCherry antibody, followed by western blotting. (**K**) PAMs were infected with PRRSV at an MOI of 0.5, and cell samples were collected for Co-IP assays to verify the interaction among IDOL, Nsp3, and SQSTM1. (**L**) Dose-dependent enhancement of the Nsp3–SQSTM1 interaction by increasing IDOL levels, analyzed by Co-IP. (**M**) Confocal microscopy analysis of IDOL, Nsp3, and SQSTM1 co-localization. Note: To minimize antibody heavy/light-chain interference in Co-IP assays, measures including cross-species antibody usage were employed, although some background interference remained.

To further confirm that IDOL-mediated Nsp3 degradation occurs via the autophagy pathway, we co-transfected cells with Nsp3 and IDOL-Flag or empty vector, followed by treatment with the autophagy inhibitors chloroquine (CQ) or Baf A1. Western blotting analysis showed that both CQ and Baf A1, which inhibit lysosomal acidification and block autophagic flux, led to marked accumulation of LC3-II and SQSTM1, along with increased levels of Nsp3 protein (Fig. 6A). These results indicate that IDOL drives Nsp3 degradation through the autophagy-lysosomal pathway. We next examined whether IDOL could induce autophagic flux using an LC3-GFP reporter plasmid. Under normal conditions, LC3 exhibited a diffuse distribution in the nucleus and cytoplasm. In contrast, IDOL overexpression caused LC3 to form distinct punctate structures in the cytoplasm, indicating the activation of autophagic flux (Fig. 6B). To further verify that IDOL promotes autophagic flux, we employed the mCherry-GFP-LC3 reporter plasmid in PK-15 cells. In the control group, green and red fluorescence were evenly distributed throughout the cytoplasm and nucleus, and their co-localization appeared yellow. In contrast, in the IDOL-overexpressing group, the fluorescence exhibited a punctate cytoplasmic distribution, with partial quenching of green fluorescence; consequently, yellow puncta decreased while red fluorescence increased, a pattern consistent with that observed following treatment with the autophagy activator rapamycin (Fig. S4A). These results demonstrate that IDOL promotes autophagic flux. A growing body of evidence indicates that selective autophagic degradation is a highly specific process mediated by cargo receptors that recognize particular substrates (22, 47). Since IDOL is not a cargo receptor, we hypothesized that it may function as an adaptor linking Nsp3 to a cargo receptor. To identify the receptor involved, we performed Co-IP assays to examine interactions among IDOL, Nsp3, and the cargo receptors NBR1, NDP52, SQSTM1, OPTN, and TOLLIP. The results revealed strong interactions between Nsp3 and both NDP52 and SQSTM1, while IDOL interacted with NDP52, SQSTM1, and TOLLIP (Fig. 6C and D). To identify which cargo receptor plays a critical role in IDOL-mediated Nsp3 degradation, we used siRNA to knock down NDP52, SQSTM1, and TOLLIP. We found that knockdown of SQSTM1, but not NDP52 or TOLLIP, largely restored Nsp3 protein levels. Furthermore, reintroduction of exogenous SQSTM1 into SQSTM1-depleted cells rescued IDOL-driven Nsp3 degradation (Fig. 6E through G). To further confirm that IDOL-driven Nsp3 degradation depends on SQSTM1, we analyzed the interactions among IDOL, Nsp3, and SQSTM1 by Co-IP. SQSTM1 showed strong interactions with both IDOL and Nsp3 (Fig. 6H and I). To verify the formation of a ternary complex, we expressed Nsp3 alone and performed Co-IP. The results indicated that Nsp3 forms a complex with endogenous IDOL and endogenous SQSTM1, and confocal microscopy confirmed clear co-localization of the three proteins (Fig. 6J and M). Subsequently, we further infected PAMs with PRRSV to verify the interaction among IDOL, Nsp3, and SQSTM1, and the results were consistent with expectations (Fig. 6K). Together, these data demonstrate that SQSTM1 participates in IDOL-mediated degradation of Nsp3. Subsequent analysis revealed that IDOL specifically enhances the interaction between Nsp3 and SQSTM1 (Fig. 6L). To define the role of IDOL in recruiting SQSTM1 to drive Nsp3 degradation, we examined their co-localization under IDOL knockdown conditions. The results showed a significant reduction in Nsp3-SQSTM1 colocalization upon IDOL depletion (Fig. S2B and S4B), consistent with IDOL facilitating this interaction.

### The UBA and LIR domains of SQSTM1 are indispensable for IDOL-mediated autophagic degradation of Nsp3

**Fig 7:**
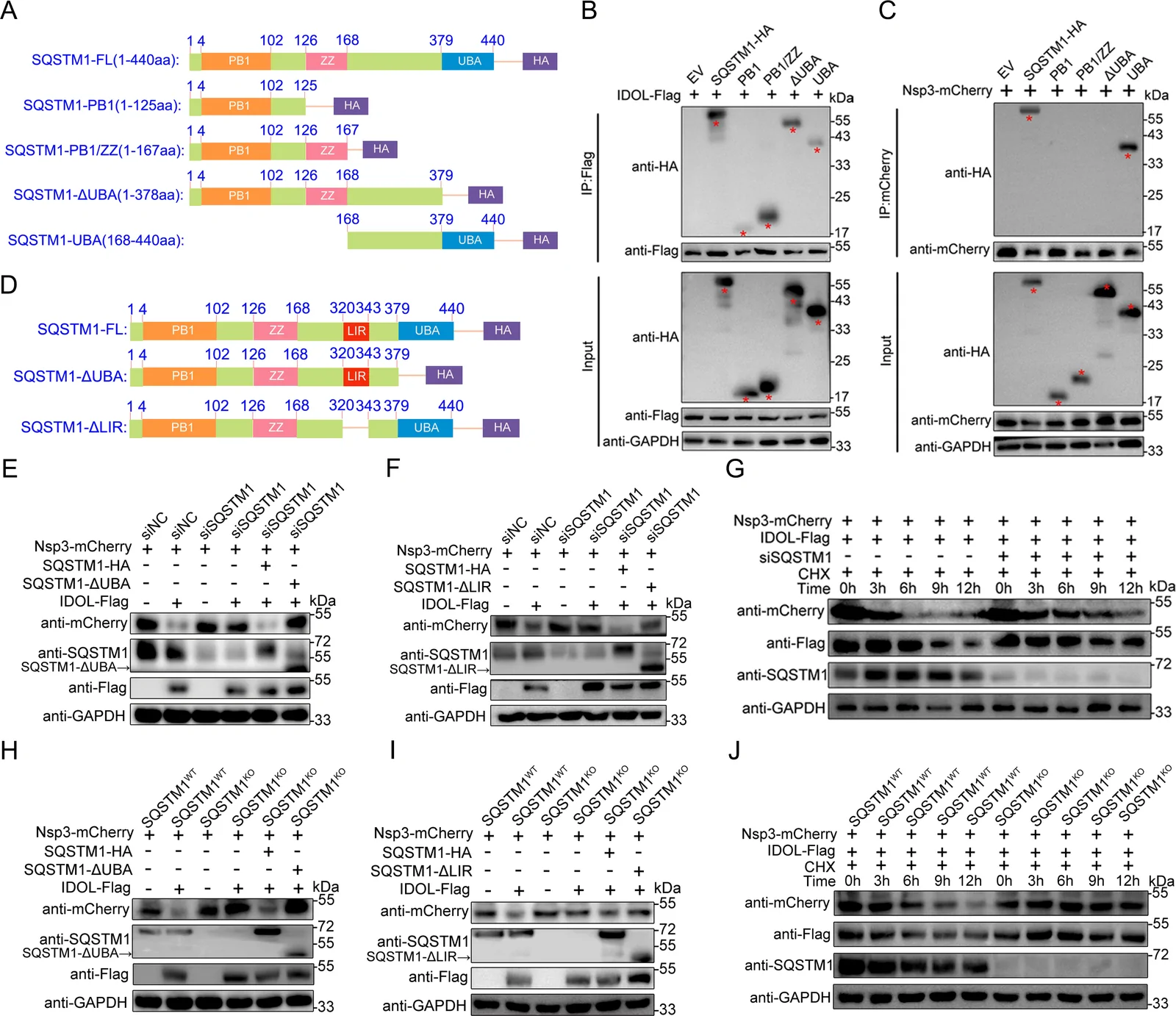
The UBA and LIR domains of SQSTM1 are indispensable for IDOL-mediated autophagic degradation of Nsp3. (**A**) Schematic of SQSTM1 truncation mutants. (**B and C**) HEK 293T cells were co-transfected with IDOL-Flag (**B**) or Nsp3-mCherry (**C**) and SQSTM1 truncation plasmids. Co-IP and western blotting were performed after 24 h. (**D**) Schematic diagrams of the SQSTM1 domain deletion mutants: SQSTM1-ΔUBA and SQSTM1-ΔLIR. (**E and F**) Functional rescue assay in SQSTM1-knockdown cells. HEK 293T cells were transfected with siSQSTM1 or NC for 24 h, followed by co-transfection with Nsp3-mCherry, IDOL-Flag, and either wild-type SQSTM1-HA, SQSTM1-ΔUBA (**E**), or SQSTM1-ΔLIR (**F**). Whole-cell lysates were analyzed by western blotting. (**G**) Degradation kinetics in SQSTM1-knockdown cells. After transfection with siSQSTM1 or NC and subsequent co-transfection with Nsp3-mCherry and IDOL-Flag, cells were treated with cycloheximide (CHX, 50 μg/mL) and harvested at the indicated time points for western blotting analysis. (**H and I**) Rescue assay in SQSTM1-knockout (KO) cells. SQSTM1-KO or wild-type (WT) cells were co-transfected with Nsp3-mCherry, IDOL-Flag, and SQSTM1-HA, SQSTM1-ΔUBA (**H**), or SQSTM1-ΔLIR (**I**), followed by western blotting. (**J**) Degradation kinetics in SQSTM1-KO cells. SQSTM1-KO or WT cells co-expressing Nsp3-mCherry and IDOL-Flag were treated with CHX and harvested at the indicated times for western blotting analysis. Note: To minimize antibody heavy/light-chain interference in Co-IP assays, measures including cross-species antibody usage were employed, although some background interference remained. The asterisk indicates the position of the target protein band.

SQSTM1 contains several functional domains, including PB1, ZZ, and a ubiquitin-associated (UBA) domain responsible for substrate recognition (48). To identify which domains mediate the interactions with IDOL and Nsp3, we constructed a series of SQSTM1 truncation mutants (Fig. 7A) and performed Co-IP assays. IDOL interacted with multiple domains of SQSTM1, whereas Nsp3 specifically bound to the UBA domain (Fig. 7B and C). Given that the UBA domain of SQSTM1 is critical for interacting with Nsp3 and that the LC3-interacting region (LIR) domain is essential for autophagic degradation, as reported (Fig. 7D) (49, 50), we hypothesized that both domains are indispensable for IDOL-mediated Nsp3 clearance. To test this, we knocked down endogenous SQSTM1 and rescued the cells with either wild-type SQSTM1 or mutants lacking the UBA or LIR domain. Re-expression of wild-type SQSTM1 restored IDOL-driven Nsp3 degradation, whereas neither domain deletion mutant could do so (Fig. 7E and F). These results demonstrate that both the UBA and LIR domains of SQSTM1 are crucial for this process. Furthermore, CHX chase assays showed that SQSTM1 knockdown significantly impaired IDOL-mediated degradation of Nsp3 (Fig. 7G). To obtain more rigorous genetic evidence and circumvent potential limitations of siRNA, such as off-target effects and incomplete knockdown, we repeated the key experiments in an SQSTM1-knockout (KO) cell line. The results regarding Nsp3 degradation efficiency were fully consistent with those obtained from the siRNA knockdown experiments (Fig. 7H through J). Collectively, these findings demonstrate that IDOL promotes the degradation of Nsp3 via the autophagolysosomal pathway by enhancing the interaction between SQSTM1 and Nsp3.

### The K101R mutation in Nsp3 enhances PRRSV replication and confers resistance to IDOL-mediated antiviral activity

**Fig 8:**
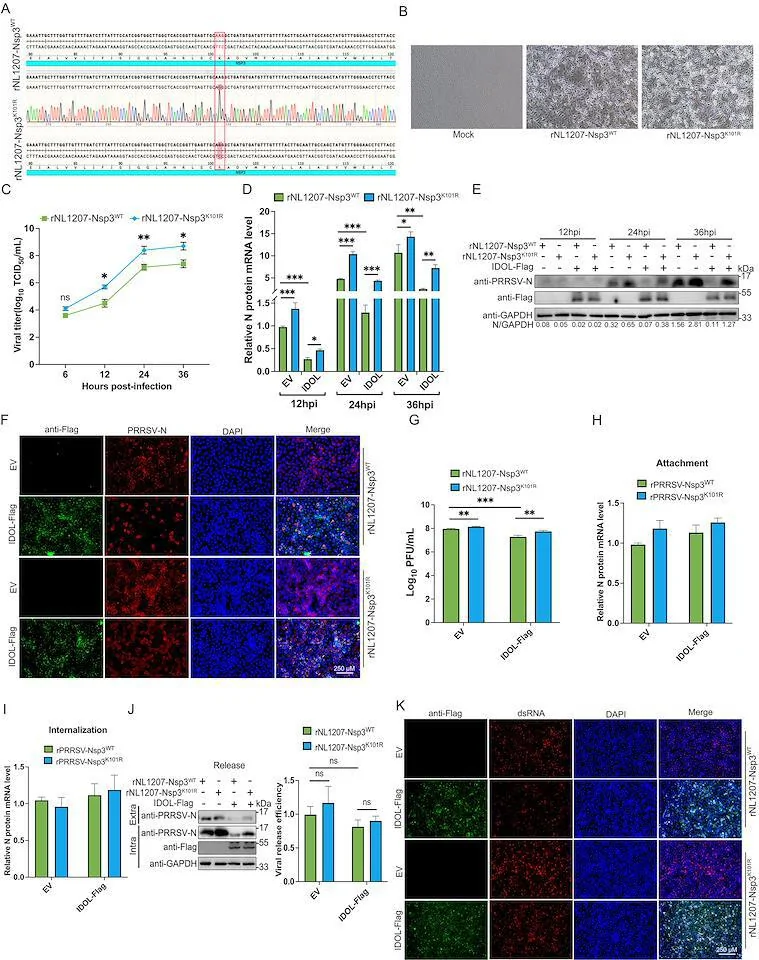
The K101R mutation in Nsp3 enhances PRRSV replication and confers resistance to IDOL-mediated antiviral activity. (**A**) Sequence alignment of infectious cDNA clones of wild-type PRRSV (Nsp3^WT^) and the mutant PRRSV (Nsp3^K101R^). (**B**) Cytopathic effects observed in Marc-145 cells infected with first-passage recombinant viruses. (**C**) Marc-145 cells were infected with recombinant viruses at an MOI of 0.01. Culture supernatants were collected at 6, 12, 24, and 36 hpi, and viral titers were determined by TCID₅₀ assay. (**D and E**) Marc-145 cells were transfected with IDOL-Flag or empty vector for 24 h, followed by infection with either PRRSV-Nsp3^WT^ or PRRSV-Nsp3^K101R^ (MOI = 0.5). Cells were harvested at the indicated time points for RT-qPCR analysis of viral RNA (**D**) and western blotting (**E**). (**F and G**) Similarly treated and infected cells were analyzed at 24 hpi by immunofluorescence assay (**F**) and plaque assay (**G**). (**H and I**) Viral attachment (**H**) and internalization (**I**) were assessed in transfected Marc-145 cells infected with PRRSV-Nsp3^WT^ or PRRSV-Nsp3^K101R^ (MOI = 10). For attachment, cells were incubated with virus at 4°C for 2 h; for internalization, cells were shifted to 37°C for 2 h after removal of unbound virus. Viral entry was quantified by RT-qPCR, measuring N gene mRNA levels. (**J**) N protein levels in both cell lysates and culture supernatants were assessed. Western blotting was used for detection, and ImageJ software was employed for subsequent quantification. (**K**) dsRNA immunostaining of transfected and infected Marc-145 cells at 18 hpi. Results are representative of at least three independent biological replicates and are presented as mean ± SD (*n* = 3). *P < 0.05, **P < 0.01, and ***P < 0.001 denote statistical significance, ensuring the reproducibility of the findings.

Based on the above results (Fig. 5F through H), the K101 site is established as critical for Nsp3 stability. To examine the functional consequence of ubiquitination at K101 of Nsp3 on PRRSV replication, we generated a point mutation (lysine to arginine) at this site within the full-length cDNA clone of the PRRSV-NL1207 strain, yielding a mutant viral cDNA construct. Following confirmation by DNA sequencing (Fig. 8A), HEK 293T cells were transfected with either wild-type or mutant infectious cDNA. Supernatants collected 48 h post-transfection were used to infect Marc-145 cells. Daily monitoring revealed distinct cytopathic effects (CPEs) in both groups approximately four days after infection, with more pronounced CPE in cells infected with the mutant virus (Fig. 8B). Culture supernatants were harvested as first-passage virus stocks, designated rNL1207-Nsp3^WT^ and rNL1207-Nsp3^K101R^, and stored at −80°C. After at least five serial passages in Marc-145 cells, multi-step growth curve analysis demonstrated that rNL1207-Nsp3^K101R^ exhibited enhanced replication efficiency compared to rNL1207-Nsp3^WT^ (Fig. 8C).

To further explore the role of the K101 site, Marc-145 cells overexpressing IDOL were infected with either rNL1207-Nsp3^WT^ or rNL1207-Nsp3^K101R^. Cells were harvested at 12, 24, and 36 hpi for assessment of N protein expression via RT-qPCR and western blotting. Both mRNA and protein levels of N protein were higher in mutant virus-infected cells than in wild-type-infected cells (Fig. 8D and E), suggesting that rNL1207-Nsp3^K101R^ evades the antiviral activity of IDOL. To corroborate this, Marc-145 cells transfected with an IDOL-expressing plasmid were infected with each virus. At 24 hpi, cells and supernatants were collected for IFA and plaque assays. IFA indicated a slightly higher infection efficiency with rNL1207-Nsp3^K101R^ (Fig. 8F), and, consistent with this, viral titers were also higher for the mutant virus (Fig. 8G).

Collectively, these results suggest that the immune escape phenotype of rNL1207-Nsp3^K101R^ is due to impaired IDOL-mediated ubiquitination and degradation of Nsp3, thereby diminishing its ability to suppress viral replication. To test this hypothesis, we evaluated the effect of IDOL overexpression on various stages of the viral life cycle for both viruses. IDOL did not significantly affect adsorption, internalization, or release of either virus (Fig. 8H through J). In contrast, during replication, dsRNA fluorescence intensity was markedly higher in cells infected with rNL1207-Nsp3^K101R^ than in those infected with wild-type virus, both with and without IDOL overexpression (Fig. 8K), indicating enhanced replication capability of the mutant and supporting our hypothesis. In conclusion, the K101 residue of Nsp3 is essential for its stability. IDOL promotes ubiquitination at lysine 101, triggering degradation of Nsp3 via the autophagy-lysosomal pathway, and ultimately inhibiting viral replication.

## DISCUSSION

**Fig 9:**
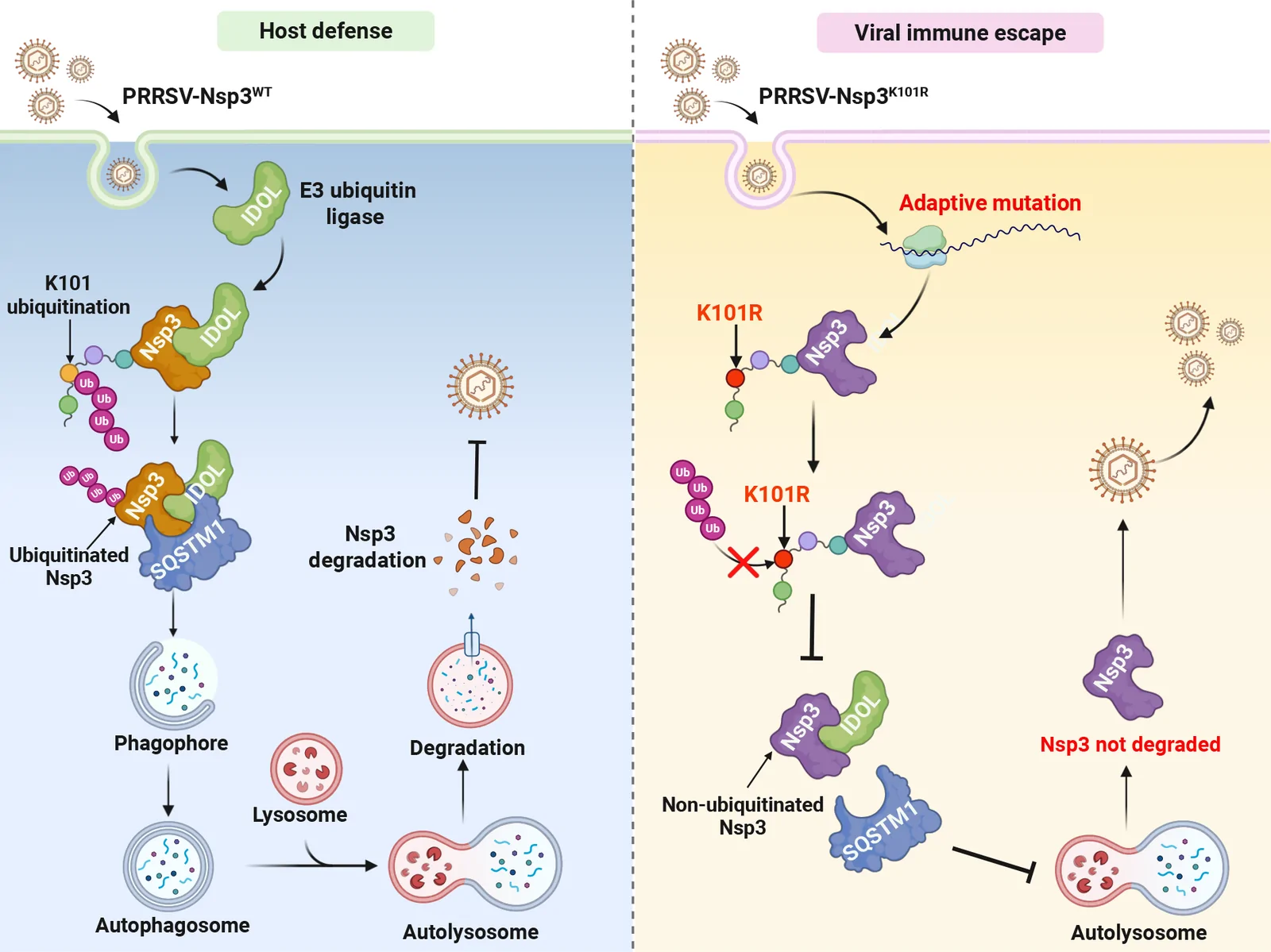
A model depicting the host-virus interface: IDOL-induced degradation of PRRSV Nsp3 versus viral escape via the K101R mutation. In terms of host defense, the E3 ubiquitin ligase IDOL orchestrates autophagic degradation of Nsp3 by catalyzing its K101-linked polyubiquitination and recruiting the selective autophagy receptor SQSTM1. As for viral immune escape, an adaptive mutation at lysine 101 of Nsp3 to arginine abrogates its ubiquitination. Consequently, IDOL-mediated degradation of Nsp3 is impaired, permitting viral gene expression and the production of infectious virions to proceed uninterrupted.

PRRSV is one of the most devastating pathogens to the global swine industry, causing massive economic losses and posing severe threats to pork supply security, food safety, and farmers' livelihoods (13, 51–53). Therefore, effective control of PRRSV is critical for the sustainable development of the swine industry and represents an important task in safeguarding national food security and social stability. In this study, we uncover a novel antiviral mechanism by which the E3 ubiquitin ligase IDOL restricts PRRSV infection, enriching our understanding of the functional interplay between IDOL and viral pathogenesis. During PRRSV infection, we demonstrate that IDOL catalyzes K63-linked polyubiquitination of Nsp3 at lysine 101 and recruits the selective autophagy receptor SQSTM1, thereby targeting Nsp3 for degradation via the selective autophagy pathway. This process effectively suppresses PRRSV replication, underscoring the potent antiviral function of IDOL (Fig. 9).

In recent years, there has been growing interest in the complex relationship between autophagy and PRRSV infection (26, 54–57). Autophagy is no longer viewed simply as a cellular process for degrading and recycling cellular components but is increasingly recognized as a central innate immune defense mechanism (58–60). The ubiquitin-proteasome system (UPS) and autophagy jointly maintain proteostasis by balancing protein degradation. Although these two pathways operate through distinct mechanisms, they frequently act on overlapping substrates and share molecular signals, particularly ubiquitin, which serves as a universal degradation tag (61). Accumulating evidence indicates a dynamic crosstalk between the UPS and autophagy, mediated in part by shared receptors and ubiquitin-tagged cargoes. SQSTM1, the most extensively studied autophagy receptor, is a typical example of this interplay: through its ubiquitin-associated (UBA) domain, it recognizes ubiquitinated substrates; its N-terminal PB1 domain mediates self-oligomerization; and its LIR domain anchors it to autophagic membranes (62). Beyond its role in proteostasis, autophagy also contributes to antiviral defense by degrading viral components and presenting antigens to activate adaptive immunity. Conversely, through long-term evolution, many viruses have evolved strategies to exploit, inhibit, or hijack autophagy, often by utilizing autophagic membranes as replication platforms to enhance propagation and persistence (56, 63). For instance, PRRSV triggers incomplete autophagy to redirect ubiquitinated antiviral proteins, whereas PEDV utilizes SQSTM1-dependent autophagic flux to promote its replication (26, 64). This dual-edged interaction has made autophagy a key focus for developing novel antiviral therapies, greatly advancing research in fundamental virology and immunology (65).

In this study, transcriptomic analysis of PAMs isolated from piglets in a challenge experiment revealed a significant upregulation of IDOL mRNA following PRRSV infection (Fig. 1). To elucidate the role of IDOL during PRRSV infection, we investigated its impact on viral replication through both ectopic expression and knockdown of endogenous IDOL. Our results demonstrate that IDOL significantly inhibits PRRSV replication, identifying it as a potent antiviral protein (Fig. 2). We next sought to determine which stage of the viral life cycle is targeted by IDOL and found that it specifically suppresses the replication phase, without affecting viral adsorption, internalization, or release (Fig. 2 and Fig. S2). E3 ubiquitin ligases regulate target protein stability and function by catalyzing substrate-specific ubiquitination. In viral infection, they function dually: the host employs them to degrade viral proteins or modulate immune signaling for defense, while viruses co-opt them to dismantle host antiviral proteins, suppressing immunity and promoting replication. Consequently, E3 ligases constitute a pivotal host-virus interface whose activity critically determines infection outcomes. Previous studies have shown that several PRRSV non-structural proteins, including Nsp2, Nsp3, Nsp5, and Nsp9, the latter possessing RdRp activity, serve as essential components of the viral RTC (15, 66–68). Impairment of these proteins often disrupts RTC assembly and affects the replication phase of the viral life cycle. We therefore examined whether IDOL modulates PRRSV non-structural proteins to inhibit viral replication. Our data show that IDOL significantly downregulates the protein levels of Nsp2, Nsp3, and Nsp7α and strongly interacts with Nsp3 (Fig. 3). Interestingly, IDOL specifically interacts with Nsp3 but not with Nsp2 or Nsp7α. This observation implies that IDOL may downregulate Nsp2 and Nsp7α through mechanisms distinct from canonical degradation pathways such as the proteasome or autophagy-lysosome system. As an E3 ubiquitin ligase, IDOL typically requires direct or indirect interaction with its substrates to exert its degradative function. Therefore, the downregulation of Nsp2 and Nsp7α may occur at the transcriptional level or via other indirect mechanisms. In contrast, the degradation of Nsp3 is mediated through the autophagy-lysosomal pathway, as demonstrated by our experimental data (Fig. 3). Further analysis revealed that the RING domain of IDOL, which possesses E3 ubiquitin ligase activity, is responsible for interacting with and degrading Nsp3 (Fig. 4). To further elucidate the mechanism of IDOL-mediated Nsp3 degradation, we examined the ubiquitination of Nsp3. We found that IDOL promotes K63-linked polyubiquitination of Nsp3 at lysine 101 in an E3 ligase-dependent manner (Fig. 5) and recruits the selective autophagy receptor SQSTM1 to direct Nsp3 for autophagic degradation (Fig. 6 and 7). Collectively, these findings underscore the crucial role of ubiquitination at lysine 101 in maintaining Nsp3 stability. During host defense against viral infection, ubiquitination plays a dual role as both an “alarm trigger” and an “effector” of viral clearance (69). Host cells employ E3 ubiquitin ligases (such as TRIM family proteins) to tag invading viral proteins with ubiquitin, targeting them for proteasomal degradation and directly impairing viral replication (42). Thus, ubiquitination is an essential defense mechanism that enables host cells to rapidly sense viruses, transmit alarm signals, and execute clearance functions (70, 71). To investigate the functional importance of the K101 site in Nsp3 during viral replication, we used reverse genetics to generate a recombinant virus, rNL1207-Nsp3^K101R^, in which lysine 101 was mutated to arginine. Subsequent experiments demonstrated that the mutant virus exhibited significantly enhanced replication capacity compared to the parental virus (Fig. 8).

In summary, the E3 ubiquitin ligase IDOL ubiquitinates Nsp3 at lysine 101 and recruits the selective autophagy cargo receptor SQSTM1, which recognizes the ubiquitinated Nsp3, thereby directing its degradation via the selective autophagy pathway. Importantly, an Nsp3 K101R mutant virus escapes IDOL-mediated degradation, validating the specificity of this antiviral mechanism and highlighting IDOL as a promising therapeutic target. (Fig. 9).

## MATERIALS AND METHODS

### Cells and viruses

African green monkey kidney cells (Marc-145) and HEK 293T cells were cultured in Dulbecco’s modified Eagle’s medium (DMEM; Gibco, 8122784) supplemented with 10% fetal bovine serum (FBS; ExCell Bio, FSP500) at 37°C in a 5% CO_₂_ incubator. PAMs were isolated from the lungs of 6-week-old PRRSV-negative piglets by lung lavage with culture medium. The harvested PAMs were maintained in RPMI-1640 medium (Gibco, 11-875-093) containing 10% FBS at 37°C under 5% CO_2_. The PRRSV-2 strains CH-1a (GenBank AY032626.1), JXA1 (GenBank FJ548855.1), WUH3 (GenBank HM853673.2), SD16 (GenBank JX087437.1), TA-12 (GenBank HQ416720.1), NL1207 (GenBank MZ399800.1), NADC30 (GenBank MH500776), and the PRRSV-1 strain GD2025 (GenBank PZ568915) were maintained in our laboratory.

### Antibodies and reagents

The rabbit anti-IDOL polyclonal antibody (47,899) was obtained from Signalway Antibody (SAB). The following reagents were sourced from Proteintech Group: rabbit anti-Flag polyclonal antibody (20543-1-AP), rabbit anti-mCherry polyclonal antibody (26765-1-AP), rabbit anti-ubiquitin (Ub) polyclonal antibody (10201-2-AP), rabbit anti-SQSTM1 polyclonal antibody (18420-1-AP), rabbit anti-NDP52 polyclonal antibody (12229-1-AP), mouse anti-GAPDH monoclonal antibody (60004-1-Ig), mouse anti-HA monoclonal antibody (66006-2-Ig), HRP-conjugated goat anti-rabbit IgG (SA00001), HRP-conjugated goat anti-mouse IgG (SA00012), CoraLite488-conjugated goat anti-mouse IgG (SA00013), and CoraLite594-conjugated goat anti-rabbit IgG (SA00013). The rabbit anti-ATG5 monoclonal antibody (CY5766) was purchased from Abways. The mouse anti-PRRSV N protein monoclonal antibody (JN0401) was acquired from MEDIAN Diagnostics (Korea). The mouse anti-dsRNA (J2) monoclonal antibody (10,010,200) was procured from Scicons (Budapest, Hungary). Protein A/G immunomagnetic beads (B23202) were supplied by Selleck Chemicals. Small-molecule compounds MG132 (HY-13259), bafilomycin A1 (BafA1; HY-100558), chloroquine (CQ; HY-17589A), rapamycin (HY-10219), and cycloheximide (CHX; HY-12320) were obtained from MedChemExpress. Z-VAD-FMK (A834991) was purchased from Ambeed.

### Plasmid construction

The full-length cDNA of IDOL was amplified by PCR from PAMs and subsequently cloned into the pCAGGS-Flag vector. Genomic DNA was extracted using a Genomic DNA Extraction Kit (OMEGA, D3396), and the full-length as well as truncated promoter sequences of IDOL were amplified by PCR using the genomic DNA as the template. These fragments were then cloned into the pGL6-TA vector (Beyotime, D2105) to generate a series of IDOL promoter luciferase reporter plasmids (M1 through M8). Plasmids encoding HA-tagged wild-type ubiquitin (Ub-WT) and its mutant variants (K6, K11, K27, K29, K33, K48, K63) were kindly provided by Prof. Gao Yulong and Dr. Zhang Tao from the Harbin Veterinary Research Institute. Vectors expressing mCherry-tagged PRRSV proteins and HA-tagged autophagy receptors (NBR1, NDP52, SQSTM1, OPTN, and TOLLIP) were maintained in our laboratory. Site-directed mutagenesis was performed via PCR on Nsp3-mCherry and IDOL-Flag to generate the Nsp3^K101R^-mCherry and IDOL^C387A^-Flag mutant plasmids. All plasmid constructs were verified by Sanger sequencing to ensure accurate insertion and mutation.

### Piglet challenge experiment

Four-week-old piglets were intramuscularly inoculated with PRRSV strain JXA1 at a dose of 2 × 10⁵ TCID₅₀/mL, while the mock-infected group received an equal volume of DMEM. The animals in each group (*n* = 3) were housed separately to prevent cross-contamination. At 14 days post-infection, the piglets were euthanized, and lung tissue was collected. PAMs were isolated by lavaging the lungs with RPMI-1640 medium supplemented with 100 U/mL penicillin and 100 μg/mL streptomycin. The isolated cell samples were subsequently sent to OE Biotech Co., Ltd. (Shanghai, China) for transcriptome sequencing.

### RNA interference (RNAi) experiment

Small interfering RNAs (siRNAs) targeting ATG5, IDOL, NDP52, SQSTM1, TOLLIP, and ZNF460, along with a negative control siRNA, were designed and synthesized by GenePharma Co., Ltd. (Suzhou, China). The specific sequences used in this study are provided in Table S2. According to the manufacturer’s instructions, the siRNAs were transfected into HEK 293T or Marc-145 cells at approximately 60% confluence using Lipofectamine 3000, and the cells were incubated for 12 h. Subsequently, the cells were transfected with other relevant plasmids for an additional 24 h.

### RNA extraction and reverse transcription quantitative PCR (RT-qPCR)

Total RNA was isolated from cultured cells using TRIzol reagent (Vazyme, R411) according to the manufacturer’s instructions. The concentration and purity of the extracted RNA were determined spectrophotometrically. Reverse transcription was performed using a commercial cDNA synthesis kit (Vazyme, R212) to convert RNA into complementary DNA (cDNA). The resulting cDNA was then used as a template for reverse transcription quantitative PCR (RT-qPCR) using SYBR Green master mix (Vazyme, Q312) on a real-time detection system. Each reaction was carried out in technical triplicate. The relative expression levels of target genes were analyzed using the comparative ΔΔCt method, with normalization to an internal reference gene. All experiments were independently repeated at least three times to ensure reproducibility and statistical reliability. Table S3 summarizes all primers used for quantitative PCR in this study.

### Dual-luciferase reporter assay

HEK 293T cells were cultured in 24-well plates at an appropriate density and co-transfected with the luciferase reporter plasmids—either the IDOL promoter reporter constructs or the pGL6-TA control plasmid—along with the pRL-TK *Renilla* internal control plasmid, using Lipofectamine 3000 reagent (Invitrogen, L300015). At 24 h post-transfection, the cells were harvested and lysed. Luciferase activity was measured using a dual-luciferase reporter assay kit (Vazyme, DL101-01) according to the manufacturer’s instructions. Firefly luciferase values were normalized to *Renilla* luciferase activity as an internal control. All transfections and luciferase assays were performed in triplicate, and the results were analyzed from three independent experiments.

### Western blotting assay

Cells were washed twice with ice-cold PBS and lysed on ice for 30 min using RIPA lysis buffer (Beyotime, P0013C) supplemented with protease inhibitor cocktail (Solarbio, P0100). The lysates were collected into 1.5 mL microcentrifuge tubes and centrifuged at 12,000 rpm for 10 min at 4°C. The supernatant was collected as the protein sample and stored at –20°C. Protein samples were separated by SDS-polyacrylamide gel electrophoresis (SDS-PAGE) and transferred onto polyvinylidene fluoride (PVDF) membranes (Merck Millipore, ISEQ00010). The membranes were blocked with 5% skim milk at room temperature for 2 h, followed by incubation with appropriate primary antibodies at 4°C overnight. After washing three times with TBST (Tris-buffered saline with Tween 20), the membranes were incubated with horseradish peroxidase (HRP)-conjugated secondary antibodies for 1 h at room temperature. Following another three washes, protein bands were visualized using an enhanced chemiluminescence (ECL) kit (MeilunBio, MA0186-3).

### Co-immunoprecipitation (Co-IP) assay

HEK 293T cells were transfected with the corresponding plasmids using Lipofectamine 3000 reagent for 24 h. After transfection, the cells were collected and lysed on ice with IP lysis buffer. Concurrently, specific antibodies were conjugated to magnetic beads by incubation at room temperature for 30 min. The beads were then washed three times with PBS containing 0.02% Tween-20. A portion of the cell lysate was reserved for whole-cell protein analysis, while the remainder was incubated with the antibody-conjugated magnetic beads overnight at 4°C or for 2 h at room temperature. Following incubation, the beads were washed three times and resuspended in SDS-PAGE loading buffer. The samples were boiled at 95°C for 10 min and subsequently analyzed by western blotting.

### Indirect immunofluorescence assay

The collected cell samples were fixed with ice-cold methanol for 15 min and blocked with 5% bovine serum albumin (BSA; Beyotime, ST2249) at room temperature for 1 h. Subsequently, the samples were incubated with specific primary antibodies overnight at 4°C. After washing with PBS, they were incubated with corresponding fluorescently labeled secondary antibodies at 37°C for 1 h. Finally, the nuclei were stained with DAPI for 10 min, and fluorescence images were captured using either an inverted fluorescence microscope (Nikon ECLIPSE Ti2) or a confocal laser scanning microscope (Leica, SP8).

### Plaque assay

The PRRSV viral stock was serially diluted 10-fold in serum-free DMEM, and each dilution was inoculated onto confluent monolayers of Marc-145 cells. After inoculation, the cells were placed in a cell culture incubator. Following a 1-h adsorption period, the viral supernatant was removed, and the cells were gently washed twice with PBS. An overlay medium, consisting of MEM (Gibco, 11,095,080) supplemented with 2% fetal bovine serum and 1% low-melting-point agarose (Sangon Biotech, A600015), was added to cover the cell monolayer. The culture plate was then inverted and returned to the incubator for continued cultivation. After 72 h, the plates were taken out and the cells were fixed with 4% paraformaldehyde, followed by staining with 1% crystal violet. The viral titer was determined based on plaque counts and expressed as plaque-forming units per milliliter (PFU/mL).

### Inhibitor treatment

For protein degradation pathway analysis, HEK 293T cells were transfected with the relevant plasmids for 12 h and subsequently treated with the proteasome inhibitor MG132 (10 μM), the autophagy inhibitors CQ (10 μM), BafA1 (1 μM), or the apoptosis inhibitor Z-VAD-FMK (10 μM) for an additional 12 h prior to cell harvest. For protein half-life determination, HEK 293T cells were transfected with the appropriate plasmids for 24 h, followed by treatment with the protein synthesis inhibitor CHX (50 μg/mL). Cells were then collected at the indicated time points to evaluate protein stability over time.

### Chromatin immunoprecipitation (ChIP) assay

The ZNF460-Myc plasmid or the empty vector control was transfected into Marc-145 cells. At 24 h post-transfection, cells were harvested and subjected to chromatin immunoprecipitation (ChIP) analysis using a commercial ChIP Assay Kit (Beyotime, P2078).

### Lentivirus packaging

Healthy, logarithmically growing HEK 293T cells were seeded into culture dishes. Once the cells reached 70%–90% confluence, a mixture of pLV2-IDOL-3×Flag, psPAX2, and pMD2.G at an appropriate ratio was incubated with transfection reagent in serum-free medium to form complexes, which were then added dropwise to the cells. At 12 h post-transfection, the medium was replaced with fresh complete medium, and cells were cultured for 48–72 h to allow lentivirus secretion. The lentivirus-containing supernatant was collected and concentrated by PEG precipitation. The concentrated virus was aliquoted and stored at −80°C.

### Construction and rescue of recombinant viruses

The full-length cDNA clone of the PRRSV-NL1207 strain (designated PRRSV-Nsp3^WT^) was used as the backbone for site-directed mutagenesis. The lysine residue (AAG) at position 101 of Nsp3 was replaced with arginine (AGG) via PCR-mediated mutagenesis, yielding the mutant construct PRRSV-Nsp3^K101R^. The mutation was confirmed by DNA sequencing prior to virus rescue. Virus rescue was performed following established protocols with slight modifications (72). Briefly, HEK 293T cells were seeded in 12-well plates one day before transfection. At approximately 80% confluence, the cells were transfected with 2 μg of the full-length infectious cDNA clone using Lipofectamine 3000. The supernatant was collected 48 h post-transfection and served as the P0 virus stock. The P0 virus was used to infect confluent monolayers of Marc-145 cells, which were monitored daily until significant CPE was observed, after which the supernatant was harvested and cryopreserved as P1 virus at –80°C. The rescued recombinant virus was then serially passaged five times in Marc-145 cells prior to its use in subsequent experiments.

### Statistical analysis

All statistical evaluations were performed using GraphPad Prism 8 software. Intergroup differences were assessed by Student’s *t*-test, and one-way or two-way ANOVA was used for multigroup analyses. Statistical significance was designated at *P < 0.05, **P < 0.01, and ***P < 0.001, with "ns" indicating nonsignificance.

## Data Availability

All data supporting the conclusions are included in this article and its supplemental materials. Additional data sets are available from the corresponding author upon reasonable request.

## SUPPLEMENTAL MATERIAL

Supplemental material mbio.01534-26-s0001.docx Figures S1 to S4 and Tables S1 to S3.

ASM does not own the copyrights to Supplemental Material that may be linked to, or accessed through, an article. The authors have granted ASM a non-exclusive, world-wide license to publish the Supplemental Material files. Please contact the corresponding author directly for reuse.
