# Supplementary material for: The E3 ubiquitin ligase IDOL suppresses PRRSV replication by targeting viral Nsp3 for SQSTM1-mediated selective autophagic degradation
Source: mBio. 2026 Sep 15;17(10):e01534-26. doi: 10.1128/mbio.01534-26 (PMC13712804; doi:10.1128/mbio.01534-26)
Supplement: Supplementary file 1 [file mbio.01534-26-s0001.docx]

Supplementary Materials for

**The E3 ubiquitin ligase IDOL** **suppresses PRRSV replication by targeting viral Nsp3 for SQSTM1-mediated selective autophagic degradation**

Jiecong Yan^1^, Yuhan Wu^1^, Huirui Xu^1^, Pengning Lai^1^, Ling Huang^1^, Zhan He^1^, Yongsheng Xie^2,^*,Chunhe Guo^1,^*

*Corresponding authors: Dr. Chunhe Guo, [guochunh@mail.sysu.edu.cn](mailto:guochunh@mail.sysu.edu.cn); Dr. Yongsheng Xie, [xysh223@163.com](mailto:xysh223@163.com)

This file includes Figures S1 to S4 and Tables S1 to S3.

**Figure S1.** PRRSV infection upregulates IDOL expression via the transcription factor ZNF460. (**A**-**D**) Time-dependent upregulation of IDOL. Marc-145 cells (A, C) and PAMs (B, D) were infected with PRRSV CH-1a at an MOI of 0.5 and harvested at 3, 6, and 9 hpi. IDOL mRNA levels were determined by RT-qPCR (A, B), and endogenous IDOL protein levels were assessed by Western blotting (C, D). (**E**) To assess promoter activity, HEK 293T cells were transfected with firefly luciferase reporter constructs containing truncated segments of the IDOL promoter (pGL6‑TA vector). (**F**) Putative transcription factor binding sites (TFBSs) in the IDOL promoter were predicted using the JASPAR vertebrate database. (**G**)To evaluate the mRNA levels of transcription factors predicted to be involved in the viral response, we infected cells with the CH-1a strain for 24 hours and then performed qRT-PCR analysis. (**H**) Marc‑145 cells were transfected with plasmids encoding KLF15, ZNF263, ZNF460, or SP40. Cells were collected 24 h post-transfection, and IDOL mRNA levels were determined by qRT‑PCR. (**I**) To further confirm IDOL regulation by ZNF460, Marc‑145 cells were transfected with siRNA targeting ZNF460, followed by qRT‑PCR analysis of IDOL expression. (**J**) To determine whether ZNF460 transcriptionally regulates IDOL, we co-transfected cells with a ZNF460 expression plasmid and a firefly luciferase reporter vector driven by the IDOL promoter. Luciferase activity was then measured to assess promoter activation. (**K**)To assess binding, we transfected Marc-145 cells with the ZNF460-Myc plasmid or an empty vector, harvested the cells after a specified period, and performed chromatin immunoprecipitation (ChIP). Results are representative of at least three independent biological replicates and are presented as mean ± SD (n = 3). *P < 0.05, **P < 0.01, and ***P < 0.001 denote statistical significance, ensuring the reproducibility of the findings.

**Figure S2**. Knockdown of endogenous IDOL in PAMs enhances PRRSV infection. (**A**) Knockdown efficiency of simIDOL-1 and simIDOL-2 in Marc-145 cells was assessed by Western blot at 24 h post-transfection, with NC siRNA as a control. (**B**) Knockdown efficiency of three porcine-specific siRNAs (sipIDOL-1, -2, -3) in PAMs was validated by Western blot at 24 h post-transfection, relative to an NC siRNA. (**C**-**E**) PAMs, transfected with sipIDOL-2 plus sipIDOL-3 or NC siRNA for 24 h, were infected with the CH-1a strain (MOI = 0.5). Viral infection was analyzed at 24 hpi by qRT-PCR (C), Western blot for N protein (E), and plaque assay (D). (**F**) Endogenous IDOL mRNA and protein expression in Marc-145 cells and PAMs under physiological conditions. (**G**) PAMs transfected as above were infected with CH-1a strain (MOI = 0.5) and collected at 6, 9, and 12 hpi for qRT-PCR. Note: The siRNA names incorporate the prefixes “sim-” (for simian/ African green monkey) and “sip-” (for porcine/pig) to indicate the species of origin. Results are representative of at least three independent biological replicates and are presented as mean ± SD (n = 3). *P < 0.05, **P < 0.01, and ***P < 0.001 denote statistical significance, ensuring the reproducibility of the findings.

**Figure S3.** IDOL promotes sustained degradation of Nsp3 during PRRSV infection. (**A**) Co-IP analysis of the interaction between Nsp3 and IDOL in lung tissues from PRRSV-infected and mock-infected piglets. (**B**) Marc-145 cells overexpressing IDOL or a control vector were infected with PRRSV CH-1a and harvested at the indicated time points (24, 36, 48, and 60 hpi). Nsp3 protein abundance was analyzed by Western blotting.

**Figure S4**. IDOL promotes autophagic flux in PK-15 cells. (**A**) PK-15 cells were transfected with the mCherry-GFP-LC3 reporter plasmid together with control or IDOL-overexpressing constructs, or treated with rapamycin as a positive control. (**B**) PK-15 cells were co-transfected with sipIDOL-2 plus sipIDOL-3 (or NC siRNA) together with plasmids expressing Nsp3 and SQSTM1, followed by confocal microscopy analysis.

**Table S1.** Virus strains utilized in the amino acid alignment analysis.

|  | Name | Genbank |  | Name | Genbank |
| --- | --- | --- | --- | --- | --- |
|  | EuroPRRSV | AY366525 |  | BJ-4 | AF331831 |
|  | SHE | GQ461593 |  | VR2332 | EF536003 |
|  | Olot/91 | KF203132 |  | XW008 | KF724404 |
|  | DV-MLV | MT311646 |  | HUN4 | EF635006 |
|  | BJEU06 | GU047344 |  | JXA1 | EF112445 |
|  | FJEU13 | KP860912 |  | TJ | EU860248 |
|  | HeB3 | MN927227 |  | WUH3 | HM853673 |
| PRRSV-1 | ZD | OP355712 | PRRSV-2 | HK13 | KF287140 |
|  | HK3 | KF287129 |  | CH-1a | AY032626 |
|  | NMEU09 | GU047345 |  | 104194 | KT257957 |
|  | FJQEU14 | KP860913 |  | NL1207 | MZ399800 |
|  | lena | JF802085 |  | NADC30 | JN654459 |
|  | WestSib13 | KX668221 |  | NADC34 | MF326985 |
|  | Tyu16 | MT008024 |  | ISU90 | KT257999 |
|  | EUGDHD2018 | MK639926 |  | QYYZ | JQ308798 |

**Table S2.** The sequences of siRNAs used in this study.

| Name | Forward sequence (5ʹ-3ʹ) | Reverse sequence (5ʹ-3ʹ) |
| --- | --- | --- |
| si *ATG5* | CCUUUCAUUCAGAAGCUGUUUTT | AAACAGCUUCUGAAUGAAAGGTT |
| si *ATG7* | AGAAGAAGCUGAACGAGUATT | UACUCGUUCAGCUUCUUCUTT |
| si m*IDOL-1* | GGCGACUGGGAAUCAUAGATT | UCUAUGAUUCCCAGUCGCCTT |
| si m*IDOL-2* | GGCUUAAACUGAGAGUCAATT | UUGACUCUCAGUUUAAGCCTT |
| si p*IDOL-1* | GAAUUAUAGAAGUUGAUUATT | UAAUCAACUUCUAUAAUUCTT |
| si p*IDOL-2* | GGCUUAAACUGAGAGUCAATT | UUGACUCUCAGUUUAAGCCTT |
| si p*IDOL-3* | GCAAUUUACGGGUAGCAAATT | UUUGCUACCCGUAAAUUGCTT |
| si *NDP52* | UUCUGUAUCAUCAUUGAACTT | UUCUUUCACUUUCAGUUCCTT |
| si *SQSTM1* | AGAUUCGCCGCUUCAGCUUTT | AAGCUGAAGCGGCGAAUCUTT |
| si *TOLLIP* | GCGUGGACUCUUUCUAUCUTT | AGAUAGAAAGAGUCCACGCTT |
| si *ZNF460* | GUUCAAUGAUGAUACAGAATT | UUCUGUAUCAUCAUUGAACTT |

**Table S3.** The primers used for qRT-PCR in this study.

| Name | Forward sequence (5ʹ-3ʹ) | Reverse sequence (5ʹ-3ʹ) |
| --- | --- | --- |
| s*CBLB* | aggcatagttcggtctccct | cttgatgctggggagaaggg |
| s*DTX3L* | actttgactgggttgccaca | atgcagttcgacgtgttcct |
| s*G2E3* | atgagcattgtgatgtccg | atgttttttggctttttgg |
| s*HECTD4* | ctggagctactctgtcccct | ctgcaaatggaggtttgggc |
| s*HERC1* | acttggccttgggttgttca | ttcacgggctgagaggaaac |
| s*LTN1* | tgcttcgtctcagcttcgag | cagccacgccaactcttttc |
| s*IDOL* | atggctaaatctaagaaatcgg | tgagttccactgcctgct |
| *sNEDD4* | ggtagctgggatggcagttt | gcgggatgtaccagtgacaa |
| *sRFWD3* | gaggagttcggcttcacaca | gctttcttgttgcactgggg |
| *sRNF4* | gtggcggggcagtaaattct | tgggcaagtgttagcgttct |
| *sRNF138* | acattgtcccctctgtcgtg | tgcgtttcctcatctagctga |
| *sRNF145* | cctctgtgtggtggcctatg | cccttcctggccgtctaatc |
| s*RNFT1* | tctcttggaattgggctgct | cgcaaaacccgtgtgaaagt |
| s*SHPRH* | ggcagcaaaggaccaagttg | actccacagctactaggggg |
| s*SMURF1* | cagacccgaggttacaccac | aagcccccgttgatgtagtg |
| s*TRAF7* | cccgcccctcctcaaaatg | ccgatgaacgtgcacccgt |
| s*TRIM21* | tccacgaagagtttgtccgg | gagtctcgacacacacccag |
| s*TRIM37* | aggtagccaaacttcgtcgg | caagccagctgagagttcca |
| s*TRIM38* | aaatgtcaagcctccgaccc | agcccaactcctgctcaaag |
| s*TRIM45* | actcaggcaagactcactgc | tcgcagagctcacagaacag |
| s*TRIM47* | caacagcctggagagtgagg | ttgagcctgcgtgtgaagag |
| s*TRIM52* | gccttacctccctacgcaaa | gtaggctctgttctcgctgg |
| s*TRIM68* | cgttgatggaagccgttgtg | actcccacatgatgctctgc |
| s*GAPDH* | ggtcaccagggctgctttta | tcttctgggtggcagtgatg |
| chl*IDOL* | gcagtatagccgtgacttg | tgcttcttttgaaacgaggt |
| *PRRSV-N* | gcaagcagcaaaagaaaaagaag | gtcggcaaactaaactccaca |
| chl*GAPDH* | tgacaacagcctcaagatcg | gtcttctgggtggcagtgat |
| *PRRSV-Nsp3* | gccttaccctgcccttga | gcctccgatggaaacaaa |
| h*ATG5* | cgagatgtgtggtttggacg | ccacaggacgaaacagcttc |
| h*ATG7* | gccagctcgcttaacattgg | catctgtgggctgctaggac |
| h*GAPDH* | ggtcaccagggctgctttta | tcttctgggtggcagtgatg |

The abbreviation "s" denotes the species Sus scrofa (domestic pig), "chl" denotes Chlorocebus sabaeus (African green monkey) and "h" denotes Homo sapiens (human).
